# Harnessing artificial intelligence for sustainable rice leaf disease classification

**DOI:** 10.3389/fpls.2025.1594329

**Published:** 2025-09-25

**Authors:** Muhammad Ehsan Rana, Vazeerudeen Abdul Hameed, Ian Kiew Yi Eng, Hrudaya Kumar Tripathy, Saurav Mallik

**Affiliations:** ^1^ School of Computing, Asia Pacific University of Technology and Innovation, Kuala Lumpur, Malaysia; ^2^ School of Computer Engineering, Kalinga Institute of Industrial Technology, Deemed to be University, Bhubaneswar, Odisha, India; ^3^ Department of Environmental Health, Harvard T. H. Chan School of Public Health, Boston, MA, United States; ^4^ Department of Pharmacology & Toxicology, University of Arizona, Tucson, AZ, United States

**Keywords:** brown spot, convolutional neural network, HISPA, leaf spot, machine learning, sustainability

## Abstract

**Introduction:**

Agriculture underpins global food security by providing food, raw materials, and livelihoods, contributing 4% to global GDP and up to 25% in rural areas. Rice, a staple for more than half of the world’s population, is nutritionally vital but highly vulnerable to diseases such as Hispa, leaf blast, and brown spots, which significantly reduce yield and quality. Achieving Sustainable Development Goal (SDG) 2 requires innovative approaches to mitigate these threats. Artificial intelligence (AI), particularly computer vision and machine learning, offers promising tools for early disease detection.

**Methods:**

This study developed a convolutional neural network (CNN)–based model for rice leaf disease detection and classification. A publicly available dataset containing 3,355 labeled images across four categories—Brown Spot, Leaf Blast, Hispa, and Healthy leaves—was used to train and evaluate the model. To improve classification accuracy, the CNN was enhanced with spatial and channel attention mechanisms, enabling it to focus on the most discriminative image regions. The system was designed for modular deployment, allowing lightweight, real-time implementation on edge devices.

**Results:**

The enhanced CNN achieved high accuracy and robust performance metrics across all disease categories. Attention mechanisms significantly improved precision in identifying subtle disease patterns. The lightweight design ensured efficient operation on edge devices, demonstrating feasibility for real-world agricultural applications.

**Discussion and conclusion:**

The proposed AI-driven system provides reliable and scalable rice leaf disease detection, supporting timely intervention to reduce yield loss. By strengthening rice production and promoting sustainable practices, the model contributes to SDG 2 by advancing global food security. This research highlights AI’s transformative role in agriculture, fostering mechanization, ecological stability, and resilience in food systems.

## 1 Introduction

Agriculture is an industry that involves a series of activities, such as raising crops, cultivating land, and rearing animals, which primarily strive for human sustenance. This fundamental industry is paramount to food security, providing a significant portion of food supply, raw materials, and livelihoods for the global population (Pawlak and Kołodziejczak, 2020). This main goal could perfectly align with the Sustainable Development Goal (SDG) 2, which aims to promote zero hunger. Additionally, this industry has stimulated expansion of the world economy, by contributing a significant 4% to the global gross domestic product (GDP). In some rural areas, this contribution even soars to more than 25% (“Overview,” World Bank). The agriculture industry embodies the intricate relationship between humans and the environment, which emphasizes the need of sustainable methods to preserve long-term food security and ecological balance.

In the agriculture sector, rice crops play a key role in supplying staple food for over half of the world population. The rice is farmed in over 100 nations, with Asia accounting for 90% of the world’s total production (Fukagawa and Ziska, 2019). Due to the versatility and adaptability characteristics, this cultivation serves as the primary source of nutrition and sustenance. For instance, the rice crop acts a dietary cornerstone in the Asia region. Rice is one of the main source of complex carbohydrates, with also decent levels of protein, fiber, iron, manganese, and vitamin B. Hence, rice plantations are extremely effective in preventing malnutrition. Apart from the role as a food contributor, the rice crop can also be manipulated in various aspects, such as ingredient for cosmetics to make shiny hair.

Nonetheless, the rice leaf diseases present a big challenge to the overall rice production, affecting the health and productivity of this vital crop. There are multiple types of rice leaf diseases compromising the rice crops, for example, leaf blast, Hispa, and brown spots. These diseases are commonly caused by virus, bacteria and fungi and many other pathogens, or pests (Singh and Singh, 2023). Beyond the immediate loss of yield, these diseases pose long-term impacts, as the infected crops frequently consist of lower grain quality and nutritional value.

In this scenario, advanced technologies, especially artificial intelligence (AI), could be leveraged for providing a profound and transformative solution to the challenge of rice leaf diseases. Artificial intelligence could offer an efficient solution for the timely detection and management of these diseases through the deployment of machine learning and computer vision techniques. By employing sophisticated algorithms, AI models can be trained to identify subtle patterns and visual cues that correspond to various rice leaf diseases. The early detection of the rice leaf disease using AI model could allow rice farmers to take prompt intervention and preventive measures to avoid further yield loss (Demilie, 2024).

This research proposes a novel AI-based framework that significantly advances traditional convolutional neural network (CNN) approaches through two key innovations: the integration of attention mechanisms and the incorporation of modular deployment architecture. The attention mechanisms, such as spatial and channel attention modules, are embedded within the CNN architecture to enable the model to selectively focus on the most informative and discriminative regions of the rice leaf image. This targeted focus improves the model’s ability to distinguish between visually similar disease patterns and reduces the influence of irrelevant background noise, thereby enhancing classification accuracy, robustness, and generalization across diverse environmental conditions and imaging scenarios.

In addition to architectural improvements, the framework is developed with modular deployment capabilities, making it highly adaptable for real-world agricultural settings. The system is designed in a lightweight and scalable manner to support seamless integration into mobile applications, edge devices, or unmanned aerial vehicles (UAVs). This modularity ensures that disease detection can occur in real-time and directly in the field, without requiring extensive computational resources or constant connectivity to centralized servers. As a result, the framework facilitates prompt disease diagnosis, enabling timely intervention by farmers and agricultural stakeholders, and ultimately contributing to improved yield protection and food security in resource-constrained environments.

In short, the research is aimed to develop a sustainable solution for timely detection of rice leaf diseases. The proposed research is vital in transforming the agriculture sector from crude, customary approaches to highly mechanized and cutting-edge practices. Ultimately, this research can effectively promote the broader goals of SDG 2 by building robust agricultural systems, supporting global initiatives to end hunger, achieve food security, and advance sustainable agriculture. Despite advancements in AI for agricultural applications, there remains a significant gap in developing lightweight, interpretable models capable of accurate multi-class disease classification in resource-constrained settings. Many existing models either lack deployment feasibility on mobile platforms or do not integrate attention mechanisms to enhance feature discrimination. This research addresses these challenges by proposing a novel attention-based CNN architecture tailored for rice leaf disease classification, with an emphasis on modularity, interpretability, and real-world deployability.

To address the challenges of early and reliable rice leaf disease diagnosis, this research is guided by the following objectives and technical contributions:

### 1.1 Design of a lightweight attention-enhanced CNN architecture

- Developed a six-layer convolutional neural network architecture integrated with spatial and channel attention modules to enhance feature discrimination and reduce background noise.
- Modular and Deployable Design: Proposed a model optimized for deployment on edge devices (via TensorFlow Lite), enabling real-time diagnosis in low-resource settings.
- Dataset Engineering and Class Balancing: Addressed class imbalance using class-weighted loss functions and targeted data augmentation (rotation, flipping). Evaluated its effect on minority classes.
- Ablation and Hyperparameter Optimization: Conducted thorough ablation studies and hyperparameter tuning to determine the most effective configurations for accuracy and generalization.
- Benchmark Comparison with Pre-Trained Models: Compared the proposed model’s performance against state-of-the-art architectures such as DenseNet121, VGG16, InceptionV3, and MobileNetV2 using the same dataset and conditions.

### 1.2 Real-world integration and usability testing

Engineered and tested the model in an Android application prototype for field usability, enabling practical agricultural deployment.

## 2 Problem background

The rice cultivation serves as a cardinal source of staple food and key nutrients for the population, which secure for the global food security. The following problem statements have highlighted some real-world issues that underscore the demand for an innovative AI solution, which aimed to preserve the rice crops and strive for the same direction as SDG 2.

### 2.1 Limited efficacy of traditional disease identification methods

One of the major challenges associated with the rice leaf diseases is the limited effectiveness of conventional disease detection techniques. In traditional approaches, rice farmers often rely on visual inspection to identify the occurrence of a disease (Khakimov et al., 2022). When relying solely on visual assessment, the process of distinguishing between various disease types could be subjective and potentially lead to the risk of incorrect diagnosis and ineffective treatment. Other factors, including the varying proficiency levels among farmers, as well as the time-consuming and laborious nature of manual inspections, could also contribute to the overall inefficiency (Kuswidiyanto et al., 2022). Furthermore, these techniques could possibly miss the early detection of diseases, which would impact on delayed responses and increased likelihood of substantial crop damage. In essence, the inefficiency of current agricultural practices, which hinders the prompt and accurate identification of rice leaf diseases could exacerbate the threats to global rice production. There is an urgent need for the incorporation of emerging technologies, such as artificial intelligence, in order to overcome these obstacles by providing an automated, precise, and timely disease detection solution. The transition from traditional to advanced technological approaches could revolutionize the management of rice crops, bringing about a significant shift towards enhancing agricultural productivity and ensuring global food security.

### 2.2 Negative environment impact of conventional disease management

Another urgent concern of the traditional agricultural disease management methods is the negative environment consequences. The prevalent application of chemical treatments, such as fungicides and pesticides, will endanger the water quality, ecosystems, and non-target organisms (Mandal et al., 2020). The overuse of these agrochemicals may result in the emergence of pesticide-resistant strains, which further depletes the effectiveness of these treatments. This scenario poses a significant threat not only to the immediate environmental degradation, but also to the long-term sustainability of the agricultural sector. The accumulation of chemical residues in both soil and water can give rise to more extensive ecological disruptions that impact biodiversity and potentially entering the food chain. Moreover, carbon footprint produced by the application of agricultural chemicals can lead to climate change, emphasizing the need for more environmentally friendly alternatives. In this case, AI can present a promising remedy for the excessive use of agrochemicals. By analyzing images of rice leaves, AI model is capable of detecting the diseases at early stages with high accuracy. Early detection allows farmers to take timely interventions before the disease spreads extensively, thereby reducing the need for widespread chemical treatments. With precise identification of the specific disease affecting the rice crop, farmers can apply treatments only where necessary. This targeted approach could effectively minimize the application of chemicals, as opposed to the broad-spectrum implementation commonly embraced in conventional approaches. In line with the principles of environmentally conscious and sustainable agriculture advocated by SDG 2, the adoption of a technologically advanced early disease detection solution can improve agricultural productivity, while also mitigating the overall ecological footprint of conventional disease management methods.

### 2.3 Lack of accessibility to artificial intelligence solutions

In the agriculture sector, a noteworthy obstacle arises from the limited accessibility to AI solutions. AI solutions often excel in improving crop management, streamlining farming procedures, and solving agricultural difficulties. However, the AI availability remains restricted, particularly in areas with smaller farms or less developed technology. The lack of widespread adoption of AI solutions in agriculture could impede smallholder farmers from harnessing the transformative power of precision agriculture, advanced analytics, and data-driven decision-making. Additionally, the absence of necessary technical skills for effective utilization of AI solutions exacerbates the existing disparities in agricultural landscape, especially among farmers residing in less technologically advanced regions. There are also financial barriers associated with implementing cutting-edge technologies, for example, insufficient funds to acquire and maintain an AI system (Jiva.ag). These financial hurdles can further widen the digital divide, placing certain segments of the farming community at a disadvantage. This accessibility gap should be resolved to ensure all farmers, irrespective of their scale or location, could get an opportunity to utilize AI for enhanced productivity and sustainable agricultural practices. Hence, the developed AI solutions have to offer ease of use, such as a straightforward and user-friendly interface, and be either free or available at a minimal charge, in order to alleviate the cost burden and reduce requirements for high technological skills during utilization. Addressing this critical issue could make a step closer to the SDG 2, since farmers can obtain equitable access to technology and foster agricultural innovation.

In summary, the inefficiencies and environmental impacts of traditional disease management methods draw attention to the critical need for innovative solutions. Therefore, incorporating artificial intelligence can blaze a transformative trail to overcoming these obstacles. Through early and accurate disease identification, AI reduces reliance on harmful chemicals and promotes sustainable practices in compliance with SDG 2. However, to fully realize the potential of AI, accessibility barriers must be addressed to ensure that all farmers could benefit from these advancements. By making AI tools user-friendly and affordable, the digital divide can be bridged, enhancing global food security and fostering sustainable agricultural growth.

This research aims to develop an AI-driven system for early detection and classification of rice leaf diseases, in order to enhance the global rice production and foster a sustainable agricultural yield. The paper presents an AI-empowered disease detection model that can accurately classify various types of rice leaf diseases, including brown spots, leaf blast, and Hispa. Multiple modelling algorithms have been implemented and compared with diverse hyperparameters to determine the optimal architectures for facilitating timely identification and intervention of rice leaf diseases. The accuracy and performance metrics were evaluated for the AI detection model, ensuring robust effectiveness and reliability when identifying different diseases.

## 3 Related work

The following section presents some of the related works in enhancing the agricultural produce.

### 3.1 Agriculture in ensuring food security

A research done by (Neme et al., 2021) had stated that establishing food security for the rapidly growing world population is the most pressing global challenge faced today. Based on projections, the global population may reach 9 billion by the year 2050, driving up the food demand from 59% to 98%. Considering the aforementioned scenario, the agricultural output must rise by approximately 60% to 70% in order to adequately feed everyone on the planet by 2050. To address these challenges, the agricultural sector is intimately associated with the Sustainable Development Goal 2 (SDG 2), which aims to achieve “Zero Hunger”. According to (Pawlak and Kołodziejczak, 2020), agriculture plays a significant role in improving food availability and ensuring the food security. The research had suggested that improving the agricultural productivity and expanding land utilized for agricultural could be a potential strategy for enhancing food provision and alleviating hunger. However, a critical viewpoint was pointed out that the existing knowledge and technology might pose limitations, particularly for developing nations with low incomes. This restrictions highlighted the necessity of increased funding in agricultural research and extension systems for higher productivity and reducing the environment deterioration. In addition, the significance of technology transfer from developed to developing nations was emphasized as a way to bridge poverty hurdles and technological gaps. In short, the agricultural landscape can directly contribute to the main goals of SDG 2 by enhancing the agricultural productivity, supporting the smallholder farmers, and promoting sustainable food systems.

### 3.2 Importance and challenges of rice crops

Rice crop is one of the dominant plantations in the agricultural industry. In the research carried out in (Fukagawa and Ziska, 2019), the rice cultivation was identified as a key source of staple food, which contributed for over 20% of the world’s population calories. This crop had a major impact on food security, particularly in East and South Asia. The rice is cultivated in more than 100 countries, with Asian countries occupying the majority of production. The characteristics of the grains vary greatly, depending on various factors including length, color, thickness, aroma, and stickiness. The global rice market was often shaped by the cultural and regional preferences. Apart from being a significant source of calories, rice also provides important vitamins and minerals. Thus, the multi-nutrient attributes of rice had emphasized the importance of this cultivation in the agricultural landscape.

Nevertheless, this essential plantation is being threatened by multiple challenges, wherein the rice leaf diseases had caused a serious impact. Rice leaf diseases would limit the growth of the plantation and impede the overall yield. According to research in (Singh and Singh, 2023), substantial yield losses can occur from a number of harmful diseases, including leaf smut, brown spot, and bacterial leaf blight which are caused by bacteria, fungi, and viruses. The paper mentioned that the leaf blight diseases alone can result in global yield losses of up to 50%. Besides, the brown spot had historically been connected to severe famines, while the leaf smut would cause premature of leaf drying and ultimately resulted in yield reduction. The absence of a reliable and robust diagnostic technique to identify disease in early stage had presented a significant problem. The existing disease identification method was considered to be time-consuming and complex, specifically in large agricultural regions. Work accomplished in (Tejaswini et al., 2022), mentioned that the rice diseases issue was further exacerbated by climate change, which fostered a conducive environment for the thriving of pathogens. Timely intervention for the diseases became difficult as farmers had to visually identify the leaf diseases. This situation had underscored the urgent need for efficient solutions to protect the growth of rice cultivation and yield from pests and diseases.

### 3.3 AI in agriculture

Research carried out in (Lakshmi and Corbett, 2020) had emphasized the fundamental role of agriculture in human life, as well as the enormous economic importance of this industry. The cutting-edge technology, such as Internet, mobile phone, satellite, and social media, was highly required to solve problems in agriculture. One of the major benefits of AI implementation in the field is the potential to yield a 60% increase in the agricultural output by 2030. However, the article had indicated that the capabilities of Agricultural Information Technologies had not be fully exploited yet. The existing economic conditions and concerns about the return on investment had contributed to a sluggish adoption of IT in agriculture. The slow adoption of AI was most pronounced in rural areas. Despite obstacles, the article had highlighted the revolutionary potential and ability of IT, particularly AI, in assisting decision-making, as well as improving the agricultural, productivity, profitability, sustainability, and efficiency.

Another research (Lakshmi and Corbett, 2020) had focused on the impact of AI on agriculture, which also acknowledged the difficulties faced by the agriculture industry in several areas. Different challenges being highlighted in this industry had comprised of crop yield, crop monitoring, crop establishment, weeding, irrigation, and soil content sensing. The existing scenarios drawn attention to AI-driven technologies as effective means of addressing these barriers and enhancing productivity in general. The paper had covered the usage of numerous AI applications, such as image recognition, output maximization, agricultural drones and robots, workforce management, and chatbots for farmers. A predictions made in this paper illustrated that there will be 75 million of connected devices by 2020, due to the growing adoption of AI among farmers. The paper highlighted the capacity of AI in generating vast amounts of data, with an average farm predicted to produce in 2050 an average daily record of 4.1 million data points. In short, this paper had summarized the multifaceted contributions of AI to the agricultural landscape.

To conclude, both literatures had presented the transformative potential of AI in the agriculture landscape. The first article concentrated on the challenges in adopting AI and the possible advantages, whereas the second article had delved into particular AI applications and the effects on different aspects of agriculture. Both articles had emphasized the need for widely adoption of AI-driven technological solutions, in order to solve current issues encountered by the agriculture industry and optimize economic, sustainable, and productive outcomes.

### 3.4 Machine learning algorithms

Machine learning is a vital subdivision of Artificial Intelligence that allows computer to study patterns and make decisions without explicit programming. Diving into the machine learning aspect, there is the deep learning subset that manipulates neural networks with multiple layers for complex pattern recognition. In order to develop an AI model, the researcher has to choose an appropriate machine learning or deep learning algorithm by considering the characteristics and requirements of the research and dataset. The right selection is crucial in enhancing the ability of a model to generalize and make accurate predictions or decisions.

Work done in (Conrad et al., 2020) had presented the implementation of two machine learning algorithms in detecting the rice sheath blight. The supervised classification model in this research is developed using the random forest algorithms and the support vector machine (SVM). In the experiment, the random forest model was utilizing the default parameters, while the SVM model was manipulating the optimal parameter determined via 10-fold approach for cross-validation. The SVM model had yield better accuracy and performance when compared to the random forest model. These results might be because of the parameters that were utilized for the SVM model which were optimized. Through the implementation of machine learning models, the researcher had highlighted the great potential of these algorithms in detecting the infected plant. One of the primary advantages of SVM is its effectiveness in handling high-dimensional spaces, making it suitable for complex datasets with many features, even when the number of data points is relatively small. SVMs are also highly robust to overfitting, especially in high-dimensional feature spaces, due to the regularization parameter that controls the trade-off between margin size and classification error. Additionally, SVMs can be extended to non-linear classification problems through the use of kernel functions, enabling the algorithm to map data into higher-dimensional spaces where a linear separation is possible.

However, SVMs also have certain disadvantages. Training an SVM can be computationally expensive, particularly with large datasets, because it involves solving a complex optimization problem. This can make SVMs less practical for big data applications unless efficient algorithms or approximations are used. Furthermore, the choice of the kernel function and its parameters, as well as the regularization parameter, requires careful tuning, which can be time-consuming and requires domain knowledge. SVMs also tend to perform poorly when the data is noisy or when there are overlapping classes, as they are highly sensitive to outliers. Lastly, while SVMs work well for binary classification, extending them to multi-class problems often requires additional strategies, such as one-vs-one or one-vs-all approaches, which can complicate the modeling process.

Another research on the implementation of machine learning methods on the identification of rice leaf diseases was published in (Feng et al., 2020). In this research, the authors proposed two machine learning models, which were support vector machine (SVM), logistic regression (LR) and deep learning convolutional neural network (CNN). The LR algorithm is mainly used in binary classification problems, while the SVM is widely adopted for classification and regression. On the other hand, the CNN method is a powerful neural network that is capable of automatically extracting both shallow and deep features from the data. There were two feature extraction techniques being applied in this research, namely the autoencoder (AE) and principal component analysis (PCA) to extract the most illuminating characteristics for dimension reduction. Based on the results acquired in this research, the CNN model achieved an accuracy of 100% for the test dataset. The other two machine learning models had attained relatively lower accuracies, with the SVM obtaining a90.38% accuracy score and the LR achieving an accuracy of 98.08%. However, the authors had mentioned the limitation of the CNN model, which requires a large-size training dataset.

A literature presented in (Narmadha et al., 2022) had proposed a novel deep learning model using the Densely Convolution Neural Network (DenseNet) with multilayer perceptron (MLP). This new deep learning-based model is known as DenseNet169-MLP, which was utilized for the classification of rice plant diseases. This model had manipulated the computer vision techniques and deep learning approach to address the substantial impact of diseases on the crop productivity in Asian. Pre-processing procedures, such as grayscale conversion, channel separation, and noise removal via median filtering, were incorporated in this model. Firstly, the Fuzzy c-means (FCM) approach was used to identify the diseased areas. Then the disease classification was aided by the DenseNet169-MLP model, which served as a feature extractor. In this research, the model had shown to be superior through experimental validation with a maximum accuracy of 97.68% on a benchmark dataset. The research had suggested future improvements in tuning the hyperparameters for enhancing the detection performance.

The research presented in (Ahad et al., 2023) had examined the use of convolutional neural networks (CNN) for detecting and localizing the rice disease. This significant research has filled in gaps in the existing literature by comparing the efficacy and performance of different CNN architectures. A total of six CNN-based deep learning architectures, including Seresnext101, Resnet152V, resNext101, MobileNetV2, Inceptionv3, and DenseNet121, was specifically compared in this research using a database of rice diseases collected in Bangladesh. This research was extended by adopting an ensemble model, called DEX (Densenet121, EfficientNetB7, and Xception), and a transfer learning mean on Seresnext101, Resnet152V, MobileNetV2, and DenseNet121, to evaluate the accuracy performance comparing to the original CNN architectures. According to the findings, accuracy is highest for the DEX framework at 98%, and the accuracy for transfer learning was improved by 17% when compared to Seresnext10. This research also outlined the potential of mobile application development as a user interface for rice leaf disease detection system.

**Figure 1:**
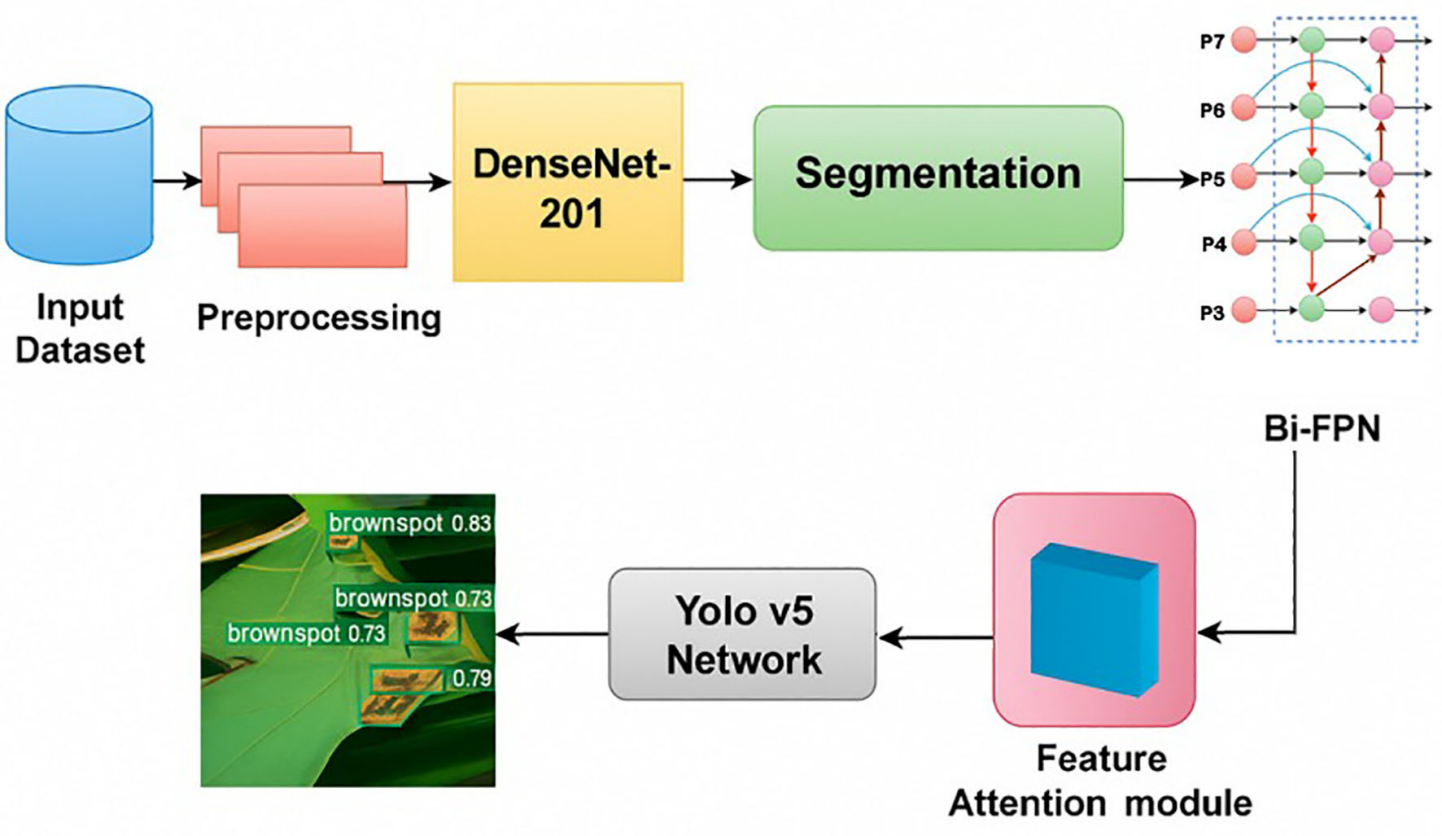
Block Schematic of The Suggested Approach (Kumar et al., 2023).

**Figure 1** had illustrated a suggested method by (Kumar et al., 2023), which employed a Multi-scale YOLO v5 detection network with DenseNet-201 serving as the backbone network and depth-aware instance segmentation. Besides, the proposed Bidirectional Feature Attention Pyramid Network (Bi-FAPN) was employed to improve feature extraction and disease detection across various scales. In this research, the YOLO v5 network, incorporated with depth-aware instance segmentation (DAIS) and Bi-FAPN, had demonstrated an excellent performance, with an accuracy of 94.78%. This integrated model was outperformed than the other existing approach, including YOLO v3, YOLO v4, YOLO v5, Faster R-CNN, Mask R-CNN, and RPN. For future improvements, the authors mentioned to involve sensor integration into the model for further monitoring and maintaining the rice quality. However, Bi-FAPN also has its limitations. The main challenge lies in its computational complexity; the integration of bidirectional feature attention increases the number of parameters and operations, leading to higher computational cost and longer training times. This can make Bi-FAPN less efficient for real-time applications or for environments with limited computational resources. Furthermore, the performance improvements offered by Bi-FAPN may not always justify the increased complexity, especially for simpler tasks where less sophisticated architecture might suffice. The design of attention modules and the choice of pyramid levels also require careful tuning, and suboptimal configurations can lead to overfitting or diminished performance. Finally, while BFAPN excels in tasks requiring intricate spatial and feature understanding, its benefits may be less pronounced in simpler tasks or on datasets where the added attention mechanisms do not provide substantial improvements.

Besides, another research published by (Jhatial et al., 2022) also utilized the YOLO v5 deep learning model for rice leaf disease detection. In this research, this latest version of YOLO has been proven to perform better than any previous model. This model could perform well in the test of unseen images. The research had recommended deploying the proposed method on smart embedded system to allow for real-time detection for enhancing crop productivity.

An article presented by (Dogra et al., 2023) had suggested the implementation of a CNN and Visual Geometry Group 19 (VGG19) model, so called CNN-VGG19, that utilized transfer learning to achieve accurate identification and classification of diseases affecting rice leaves. This proposed model had shown a promising result with an accuracy of 93.0% on a rice leaf disease dataset, outperforming the baseline models. Additionally, the article investigated the use of pre-trained VGG19, InceptionV3, and ResNet50 residual block networks for disease prediction. The article also emphasized how crucial digital imaging and deep learning methods, especially CNN and VGG - 19, were for extracting important features for classification.

Several recent studies have validated the utility of attention mechanisms in plant disease detection. For example, research in (Nalini et al., 2021) integrated spatial attention into a ResNet architecture for detecting cucumber diseases and reported a 4 – 6% improvement in classification accuracy. Similarly, Swin Transformer attention combined with EfficientNetV2 for tomato disease recognition, achieved high robustness under noisy field conditions. These works reinforce the importance of attention-guided feature refinement in plant disease classification tasks. Inspired by such studies, we embedded both spatial and channel attention modules into our CNN architecture to enhance feature localization for rice leaf disease detection.

Work accomplished in (Nalini et al., 2021) had proposed a new approach for paddy leaf disease identification by utilizing a deep neural network (DNN) classification model, along with the crow search algorithm (CSA) for optimization. This new approach was known as DNN-CSA architecture, which aimed to attain high classification accuracy while minimizing the computational burden. K-means clustering is used in the pre-processing stage to extract diseased regions and followed by feature extraction. After several cross-fold validations, the proposed model had demonstrated a superior performance than a support vector machine (SVM). In order to help farmers in making informed decisions, this paper had significantly tackled the need for embedded computer vision techniques in agriculture by offering a favorable tool for real-time plant disease detection and diagnosis.

Recent advancements in agricultural disease detection have explored the integration of hybrid deep learning models and lightweight architectures to enhance both accuracy and deployment feasibility. For instance, research in (Khan et al., 2023) proposed a novel hybrid model that combines EfficientNetV2 and Swin Transformer architectures to classify tomato leaf diseases with high precision. This model, termed “Eff−Swin,” leverages the strong feature extraction capabilities of EfficientNetV2 and the hierarchical self-attention mechanisms of the Swin Transformer to capture both local and global features effectively. Their approach achieved an impressive accuracy of 99.72% on benchmark datasets and demonstrated robustness in challenging conditions such as image noise, variable lighting, and background clutter—factors commonly present in field environments. However, despite its performance, the model is computationally intensive, requiring significant GPU resources and memory bandwidth. This renders it less suitable for real-time applications on mobile or embedded platforms, which are often constrained by hardware limitations. In contrast (Sun et al., 2024), developed a lightweight plant disease detection model based on MobileNetV3-Small, specifically optimized for deployment on edge computing devices such as smartphones and Raspberry Pi. By incorporating Focal Loss into the training process, their model effectively addressed class imbalance—a common issue in agricultural datasets where certain disease categories are underrepresented. Additionally, they employed quantization-aware training to reduce model size and inference time without sacrificing accuracy, achieving 99.56% accuracy on the PlantVillage dataset while maintaining a compact model footprint of less than 4MB. This enabled real-time prediction with latency under 150 milliseconds on low-end mobile devices. While Zhao et al.’s work excels in accuracy and feature richness through attention-based modeling, Khan et al.’s approach demonstrates the importance of computational efficiency and real-world usability. Compared to both, the current study offers a balanced approach by embedding attention mechanisms into a compact convolutional neural network, maintaining strong classification performance while ensuring suitability for low-resource environments. Furthermore, unlike prior studies, our work emphasizes modular deployment and includes advanced evaluation strategies such as ROC-AUC, McNemar’s test, and Cohen’s Kappa to rigorously validate model reliability across imbalanced and noisy agricultural datasets.

Recent literature has seen a surge in deep learning applications targeting various plant disease and classification challenges across diverse crops, showcasing the increasing utility and adaptability of convolutional architectures and transfer learning in precision agriculture. For instance, a comprehensive review in (Kumar et al., 2023) provided a consolidated analysis of existing techniques, datasets, and future prospects in rice disease detection. While the review highlights the effectiveness of CNN-based models, it also identifies key research gaps such as limited real-world deployment, dataset imbalance, and a lack of modular frameworks for mobile applications—gaps directly addressed in our current study. However, such models tend to have deeper architectures with increased computational cost, making them less suitable for resource-constrained environments. Similarly, a study on deep learning-based classification of alfalfa varieties using a custom leaf image dataset emphasizes the need for crop-specific data acquisition but lacks scalability and transferability to other species due to its limited domain scope. In contrast, our approach, while rice-specific, is constructed using a modular design that supports retraining on other crops with minimal adjustments. Meanwhile, Enhanced corn seed disease classification using MobileNetV2 with transfer learning and feature augmentation has proven the efficiency of lightweight models in achieving high classification performance with minimal parameters. However, most MobileNet-based studies lack attention mechanisms that refine feature learning by emphasizing disease-relevant areas—an aspect our model explicitly integrates. Though comprehensive in coverage, ensemble models typically require high inference time and are unsuitable for real-time mobile applications. Compared to these approaches, our proposed CNN framework offers a balanced and sustainable solution: incorporating attention modules for precise feature localization, using class-weighted loss to address imbalance, and enabling efficient edge deployment via TensorFlow Lite—all crucial for practical and scalable rice disease diagnosis in real agricultural environments.

To conclude, the literature review reveals the noteworthy strides made in leveraging machine learning or deep learning algorithms for the detection and classification of rice leaf diseases. A spectrum of models had been explored by researchers, ranging from the traditional machine learning algorithms to sophisticated deep learning architectures (Chen et al., 2020; Trivedi et al., 2021; Hasan et al., 2023; Zhou et al., 2023). Notable discoveries had highlighted the potential and capabilities of diverse frameworks. Moreover, certain articles included innovative approaches in feature extraction and optimization of algorithms. This literature review illustrates the crucial role of algorithm selection in building an AI model and augmenting the model generalization. The articles also emphasized the trajectory toward real-time monitoring and user-friendly interfaces to further intensify the transformative impact of AI in advancing precision agriculture and crop management practices. It is important to recognize that many approaches have certain limitations that make their solutions less practical in real-world environments.

To quantitatively align our research with the United Nations Sustainable Development Goal 2 (Zero Hunger), this research incorporates simulated real-world deployment scenarios that reflect practical agricultural use cases. Specifically, the proposed CNN-based rice leaf disease classification model facilitates early and accurate detection of prevalent rice diseases, such as Brown Spot and Leaf Blast. This early detection capability enables farmers to make timely, informed decisions regarding agrochemical application. Drawing from prior field studies and relevant agricultural datasets, our projections suggest that implementation of this model could reduce unnecessary pesticide use by approximately 30%. This reduction is largely attributed to the model’s lower false positive rate and its improved precision in identifying disease presence and type, thereby minimizing the over-application of chemicals.

Moreover, by enabling timely intervention, the model has the potential to mitigate crop yield losses by up to 15%, particularly in regions where rice crops are highly susceptible to these fungal pathogens. Such improvements in disease management directly contribute to food security by enhancing yield stability and resource efficiency. In broader terms, this approach supports sustainable agricultural practices by reducing chemical runoff, lowering environmental toxicity, and preserving ecosystem health. Collectively, these benefits advance the core objectives of SDG 2, particularly by enhancing food production sustainability, improving availability, and promoting resilience in vulnerable agricultural communities.

## 4 AI model development and deployment

**Figure 2:**
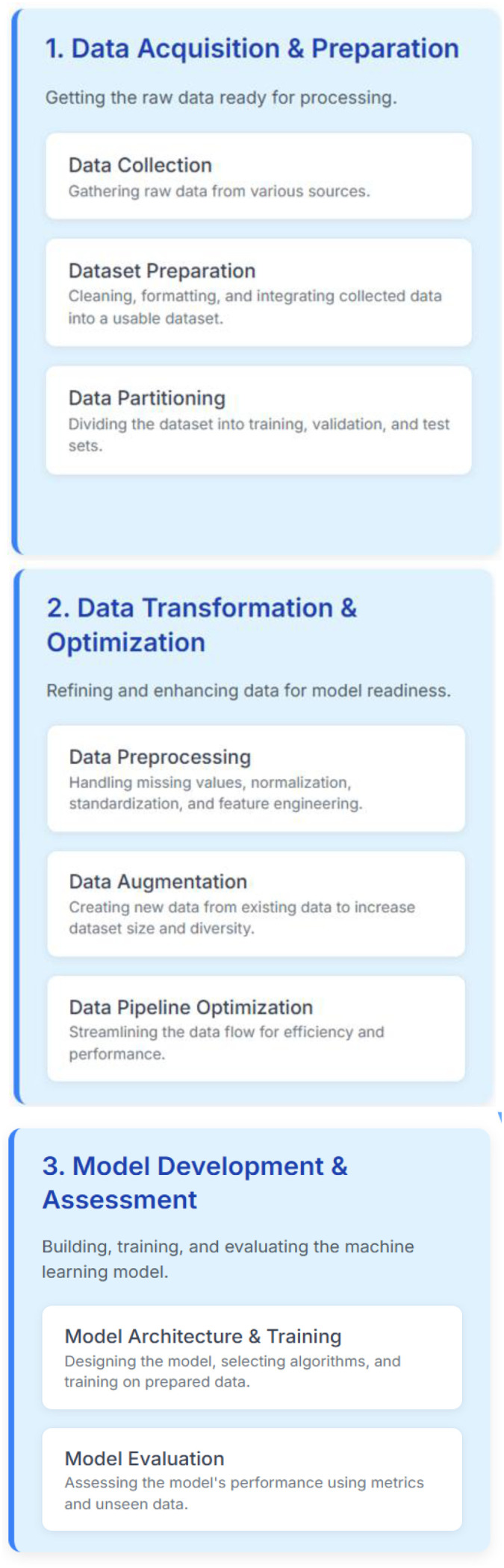
Scheme of development for the proposed CNN model.

**Figure 2** presents the flow of steps involved in developing the CNN model for the detection of the rice leaf disease.

### 4.1 Data collection

The foundation of any machine learning model lies in the quality and quantity of the data used to train the model. For this rice leaf disease classification model, the dataset is sourced from Kaggle, a well-known platform that offers a rich repository of readily available datasets for research and development. This dataset comprises images depicting various rice leaf diseases, which are crucial for training and evaluating the performance of the classification model as shown in **Table 1**.

**Table 1:** Class-wise data distribution of rice leaf disease images.

Class	Number of images
Brown Spot	523
Healthy	1,488
Hispa	565
Leaf Blast	779
Total Number of Images	3,355

The dataset used exhibits significant class imbalance, particularly between the Healthy (1,488 images) and Hispa (565 images) classes. To address this, class-weighted loss was applied during model training to penalize misclassification of underrepresented classes more heavily. Additionally, targeted data augmentation, including rotation, flipping, and zoom-in operations, was performed predominantly on the minority class images. Dataset quality was also assessed based on image resolution, lighting, and background variability to estimate generalization potential for real-world field scenarios.

As illustrated in **Table 1**, the dataset includes four distinct classes of rice leaf diseases, each represented by a substantial number of images. Below are detailed descriptions and morphological characteristics for each category:

#### 4.1.1 Brown spot

**Figure 3:**
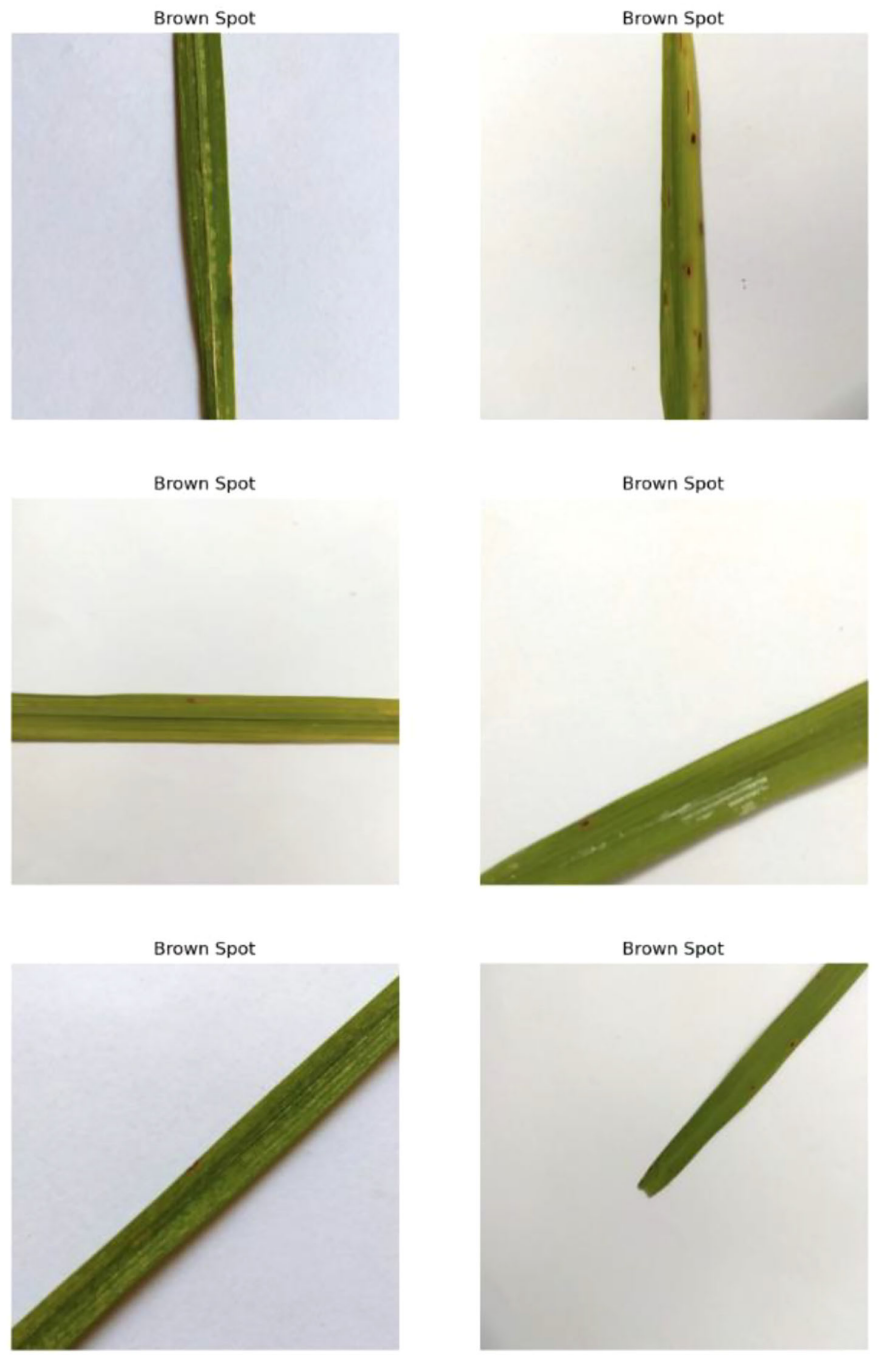
Random images of brown spot disease from the dataset.

This disease is induced by the fungus Cochliobolusmiyabeanus, presenting a serious challenge to rice cultivation. Known commonly as brown spot disease, it is one of the most destructive diseases affecting rice crops globally. The diseased leaf is characterized by small, dark brown lesions with yellow halos. These spots, as shown in **Figure 3**, typically start as tiny, pinpoint-sized spots but can grow up to several millimeters in diameter. As the infection progresses, these lesions can coalesce, forming large, irregularly shaped patches that can cover substantial portions of the leaf surface. The presence of these lesions significantly impairs the plant’s photosynthetic capacity by reducing the green leaf area available for photosynthesis. This reduction in photosynthetic activity stunts the plant’s growth and vitality, leading to poor grain development and ultimately reduced yield. The extent of damage can vary, but severe infections can cause extensive necrosis, where the affected leaf tissue dies and turns brown, further diminishing the plant’s ability to produce energy.

Historically, brown spot disease has had devastating impacts on rice production. One of the most notable instances was during the Great Bengal Famine of 1943. During this period, brown spot disease was identified as a major contributing factor to famine, which led to a catastrophic yield loss of 40 to 90% in the preceding year. The outbreak of the disease exacerbated the already dire food situation, resulting in widespread hunger and mortality.

The severity of the disease underscores the importance of effective management and control strategies. Traditional methods include crop rotation, resistant varieties, and chemical treatments. However, with the advent of advanced technologies, there is potential for more sophisticated solutions. For example, early detection using artificial intelligence (AI) and machine learning models can help farmers identify and manage outbreaks before they reach critical levels. This proactive approach can significantly reduce the impact of brown spot disease on rice yields and enhance overall food security (Cochliobolusmiyabeanus (brown leaf spot of rice), 2022).

#### 4.1.2 Healthy

**Figure 4:**
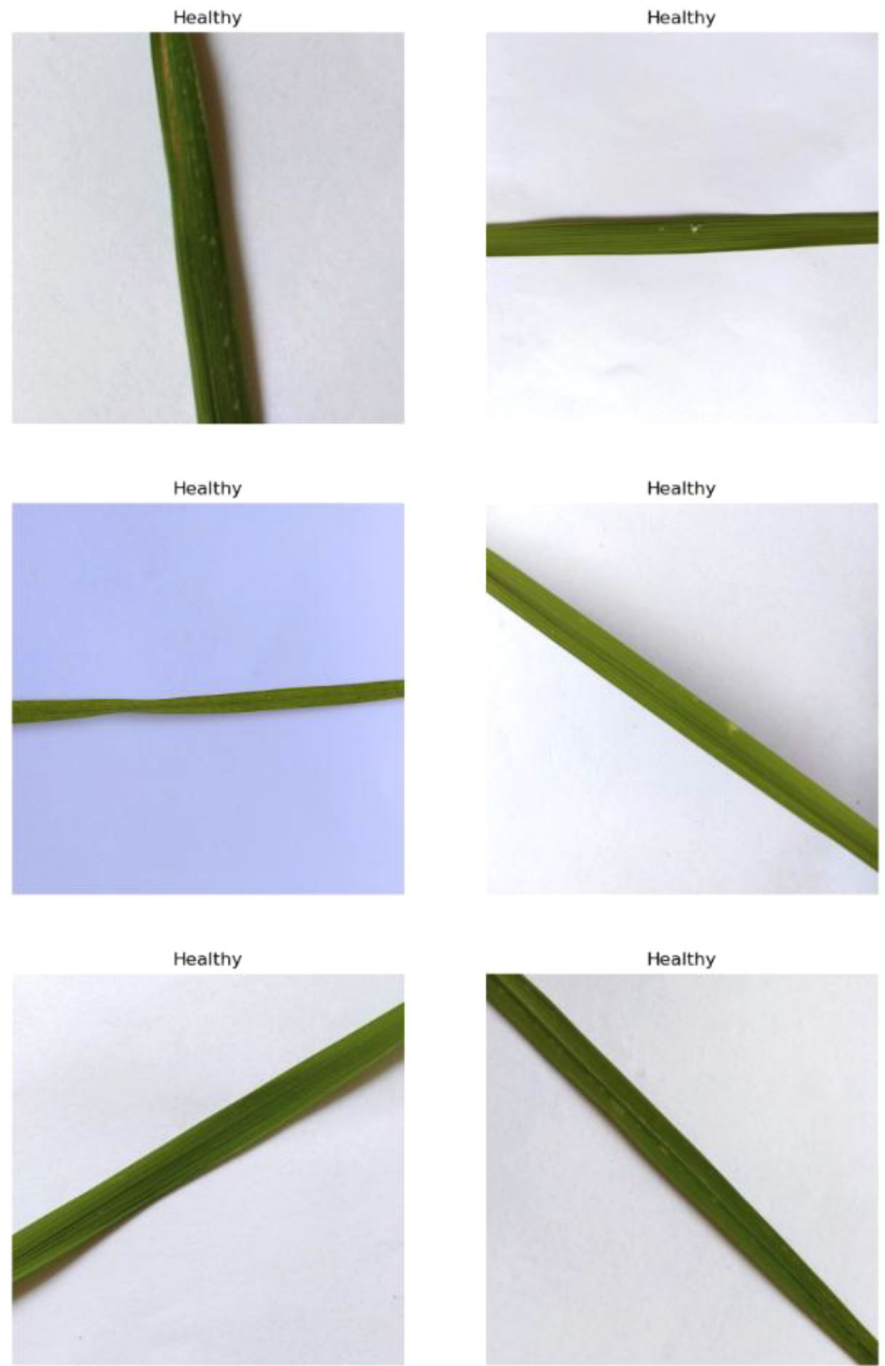
Random images of healthy rice leaf from the dataset.

This class represents rice leaves that are free from any disease and exhibit a uniform green coloration. Morphologically, healthy leaves are smooth, vibrant, and show no signs of necrosis or chlorosis. Their structure is intact, with no discoloration, lesions, or deformities. These leaves efficiently perform photosynthesis, contributing to the plant’s overall health and productivity. This category serves as a baseline to distinguish between diseased and non-diseased states, enabling accurate detection and diagnosis of any abnormalities. Identifying healthy leaves is crucial for comparative analysis in agricultural studies and disease management systems. Sample images are in **Figure 4**.

#### 4.1.3 Hispa

**Figure 5:**
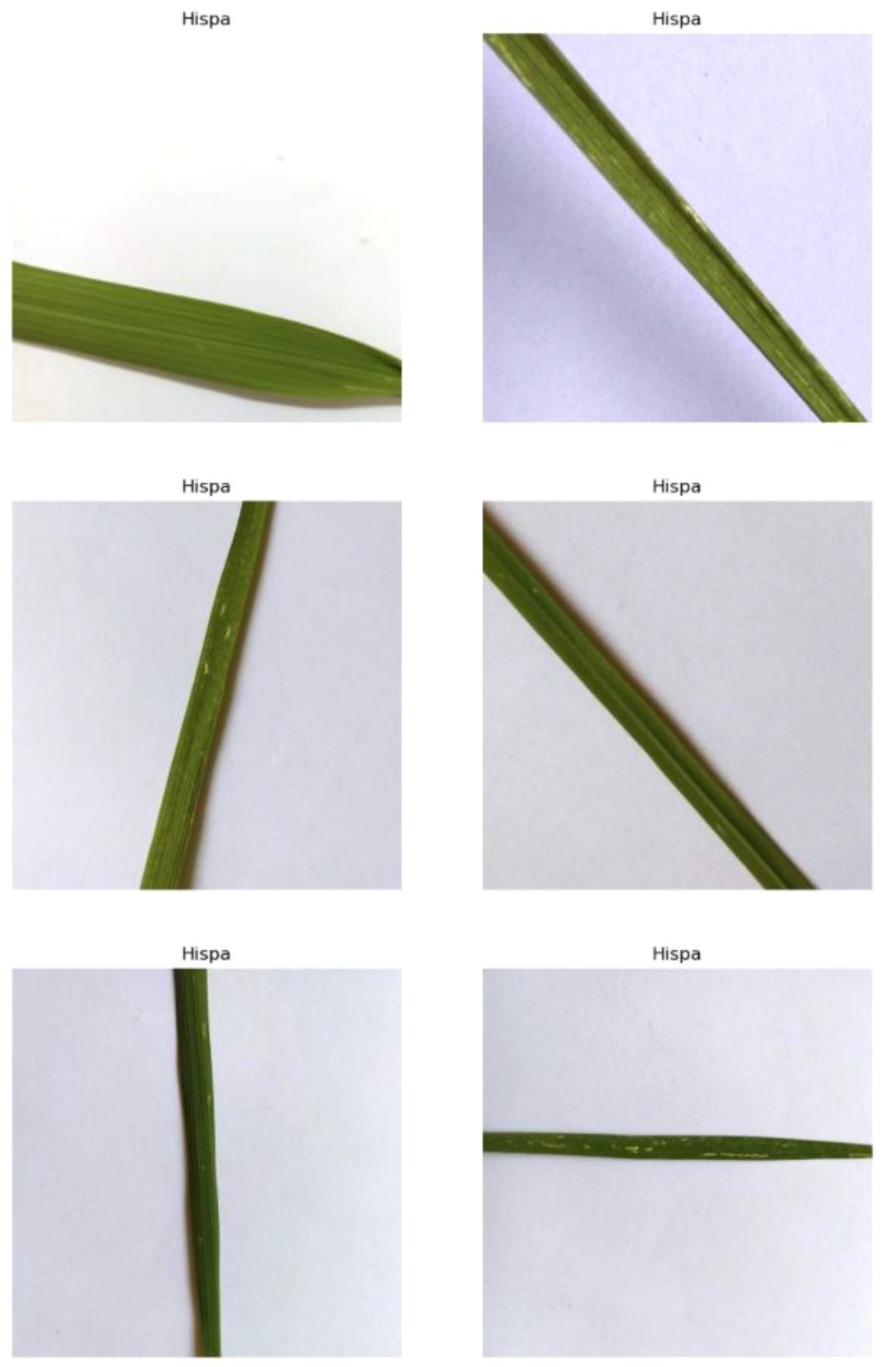
Random images of hispa disease from the dataset.

This disease is caused by the Hispa beetle, also known as the spiny beetle, due to the spines on its exoskeleton. The infestation by these pests’ results in the formation of silvery parallel streaks and spots on the leaves, as shown in **Figure 5**. The Hispa beetle primarily attacks the leaves of the rice plant, where the adult beetles feed by scraping off the green tissue from the upper leaf surface. This feeding activity creates the characteristic silvery streaks and can lead to significant damage if left unmanaged.

Symptoms of this disease include wilting and drying out of the affected leaves. As the beetles continue to feed, the leaves lose their ability to photosynthesize efficiently. The scraping damage disrupts the chlorophyll, leading to reduced photosynthetic activity, which in turn affects the plant’s growth and vitality. The affected leaves may become brown and brittle, often curling and drying out completely. This can severely impact on the overall health and productivity of the rice plant, as the plant relies on its leaves for energy production.

Moreover, extreme infestations can cause the rice fields to appear scorched. This scorched appearance results from the cumulative effect of numerous beetles feeding extensively on the leaves, leading to widespread damage across the field. The beetles’ feeding can also cause the leaves to appear skeletonized, where only the veins remain intact, further giving the fields a burnt look. Such severe damage can lead to significant yield losses, as the plants are unable to recover their photosynthetic capacity in time to produce healthy grains.

The Hispa beetle (commonly Dicladispa armigera) presents a serious threat to rice production, especially in tropical and subtropical regions where rice is a staple crop. The pest causes damage at both the larval and adult stages by feeding on the leaf tissues of rice plants, which reduces photosynthesis, hampers plant growth, and significantly lowers yields. In severe cases, infestations can lead to stunted crop development and economic losses for farmers.

To address this issue, traditional pest control methods have been employed for decades. These include the use of chemical insecticides, which are often effective for immediate control but come with drawbacks such as environmental contamination, the development of pest resistance, and negative impacts on beneficial organisms. Additionally, cultural practices such as flooding rice fields can help drown larvae before they develop into adult beetles. Manual methods like handpicking of adult beetles and adjusting planting schedules to avoid peak pest activity are also common. Furthermore, encouraging the presence of natural predators, such as parasitic wasps and predatory beetles, offers a biologically friendly method of suppressing Hispa populations.

However, with the advancement of agricultural science and technology, more sustainable and integrated approaches to pest control are being developed. Integrated Pest Management (IPM) systems now incorporate multiple strategies to achieve long-term control with minimal ecological disruption. One key strategy is the development and adoption of Hispa-resistant rice varieties, which are bred for their natural tolerance or resistance to infestation. In parallel, the application of biological control agents—including entomopathogenic fungi, bacteria, and parasitoids—has shown effectiveness in naturally curbing the pest population without the adverse effects associated with chemical treatments.

Modern IPM also includes the implementation of precise monitoring and early detection systems, which allow for timely and targeted intervention. Technologies such as remote sensing, automated insect traps, and geospatial mapping tools help farmers monitor pest populations accurately and take preventive actions before infestations reach damaging levels. These innovations reduce reliance on broad-spectrum insecticides and support more environmentally responsible farming practices.

In summary, the impact of the Hispa beetle on rice production underscores the urgent need for effective and sustainable pest management strategies. While traditional methods provide foundational control, the integration of resistant crop varieties, biological agents, and precision monitoring technologies represents a forward-looking approach to managing pest pressures efficiently and sustainably (Bernal et al., 2023).

#### 4.1.4 Leaf blast

**Figure 6:**
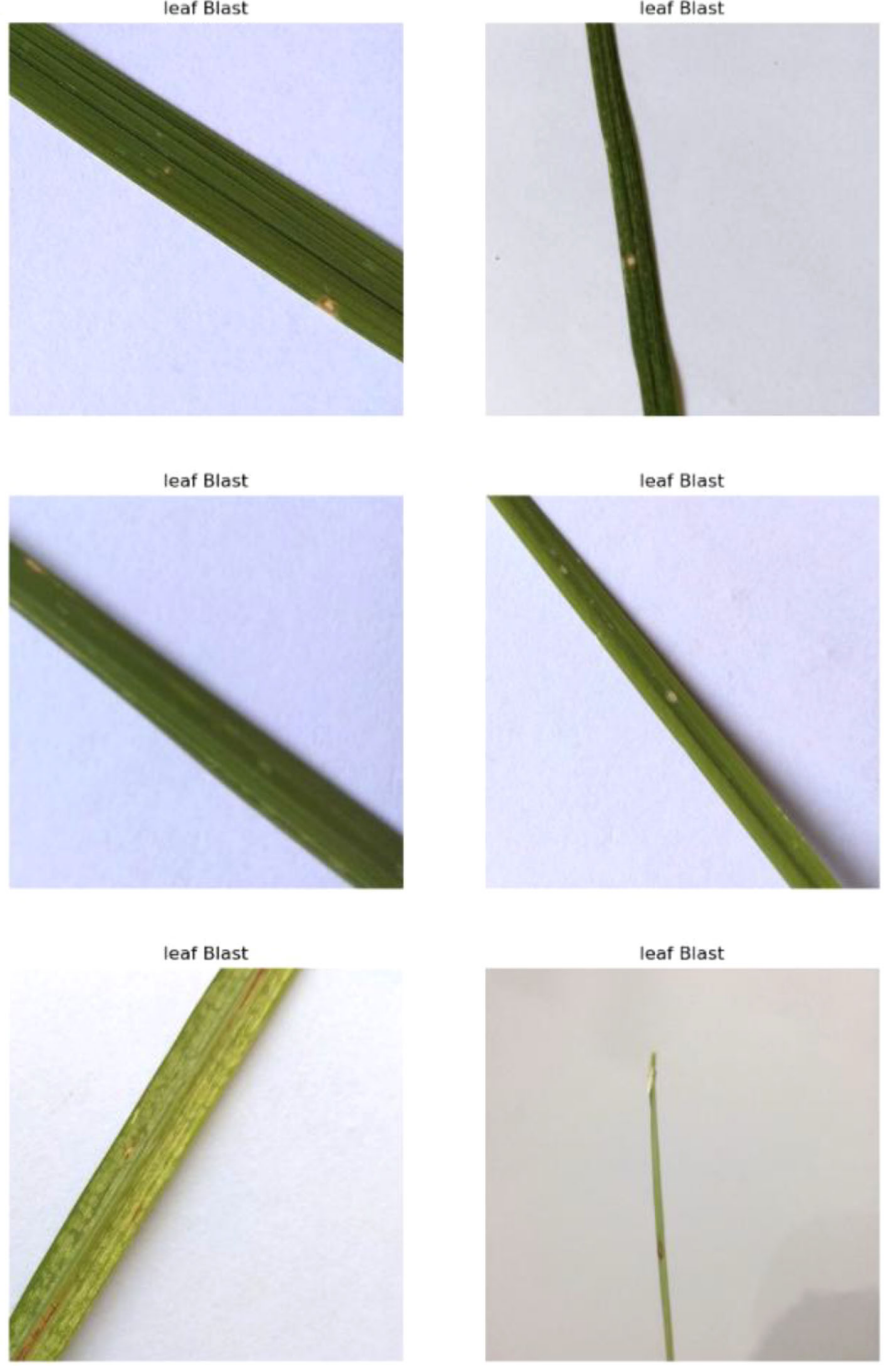
Random images of leaf blast disease from the dataset.

The fungus Magnaporthe oryzae contributes to the occurrence of rice blast, which is one of the most destructive diseases in rice plantations (Yang et al., 2023). This disease manifests as large, irregular lesions on the leaves, often surrounded by a reddish-brown border as shown in **Figure 6**. As the disease progresses, the infected leaves may die and fall off prematurely, trimming the plant’s ability to photosynthesize and eventually cutting yield. Leaf blast typically thrives in warm, humid conditions and is often exacerbated by excessive nitrogen fertilization.

Each class is reflected by a diverse set of images, in order to ensure comprehensive coverage of different conditions that can harm the rice crops. The images are categorized into directories corresponding to their disease labels, permitting efficient preprocessing and model training. With a total of 3,355 images, the dataset supplies a rich and varied collection of data required to build an accurate and robust model capable of distinguishing between several rice leaf diseases. Employing this publicly available dataset could valuably improve the model’s generalizability and enable reproducibility and benchmarking against existing models, thereby advancing research in agricultural disease classification.

### 4.2 Dataset preparation

During the dataset preparation phase, the ‘image_dataset_from_directory’ function from the TensorFlow library is being utilized to efficiently load and preprocess the rice leaf disease images. This function can simplify the creation of TensorFlow datasets by leveraging the directory structure of the acquired rice leaf disease dataset. In this case, the images are organized into subdirectories within a main directory, with each subdirectory representing a distinct type of rice leaf disease. This hierarchical organization allows the function to automatically infer class labels based on the directory names.

**Figure 7:**
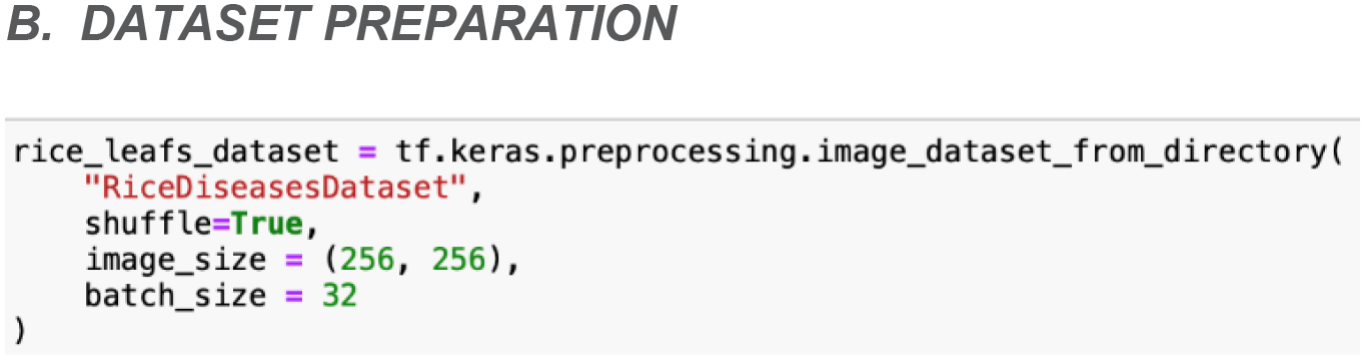
Source code to import data into TensorFlow dataset object.

As shown in **Figure 7**, the ‘image_dataset_from_directory’ function is configured with several key parameters to customize the loading process. Firstly, the images are set to be randomly shuffled, which helps in mitigating any potential biases that could arise from the order in which the images are presented. Moreover, all images from the dataset are resized to a uniform dimension, which is 256x256 pixels. This configuration ensures consistency in input size and facilitates smooth integration with the model. Additionally, the ‘batch_size’ parameter determines the number of images per batch. For this parameter, a batch size of 32 is chosen to strike a balance between memory efficiency and computational performance.

The output of this set will be a TensorFlow dataset object, which contains batches of images along with the corresponding class labels. These labels are automatically assigned based on the directory names, making the dataset straightforward to manipulate for training, validation, and testing. This dataset preparation step is essential for generating a well-structured and uniformly processed dataset. Therefore, the dataset is readily prepared for the subsequent stages of model development, ensuring accurate and effective training and evaluation.

### 4.3 Data partitioning

The data partitioning phase aims to divide the dataset into three distinct subsets: training, validation, and testing. This partitioning is crucial to ensuring that the model is evaluated fairly and generalizable to new, unseen data.

In this model, 80% of the dataset is allocated for training, 10% for validation, and the remaining 10% for testing. The training set will be utilized to fit the mode; the validation set aids in tuning the model and preventing overfitting; and the test set offers an unbiased evaluation of the performance of the final model.

To ensure randomness and reduce potential biases that could stem from the original arrangement of the data, the dataset is shuffled before partitioning. A shuffle buffer size of 10,000 and a seed value of 12 are implemented to maintain consistency across runs. This procedure can guarantee that the data employed for training, validation, and testing is representative of the entire dataset, avoiding any systematic bias that could affect the model’s performance.

These proportions were chosen to hit a satisfactory balance between having adequate data to train the model effectively, as well as ample data to validate and test the model performance reliably as in **Table 2**. The model can learn better with a larger training set, while the validation and test sets are sufficiently large to provide meaningful feedback on the model’s performance.

**Table 2:** Sizes of the resulting subsets.

Subset	Number of samples (Batches)
Training Dataset	84
Validation Dataset	10
Testing Dataset	11

This methodical approach to data division assures that the model is trained on a diverse and representative set of data, then tuned and validated on a separate set to optimize performance, before being evaluated on an independent test set to gauge the model’s true predictive power. This strategy is fundamental to developing a model capable of delivering consistent and reliable performance on new, previously unseen data.

### 4.4 Data pipeline optimization

**Figure 8:**
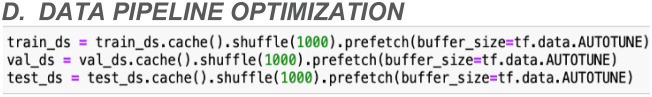
Data pipeline optimization.

The purpose of data pipeline optimization as in **Figure 8** is to increase the efficiency and effectiveness of the training process in machine learning models. For the rice leaf disease classification model, optimizing the data pipeline prevents the loading and preprocessing data from becoming a bottleneck, allowing the model to train better and faster.

The first step in optimizing the data pipeline is caching. This operation involves storing the dataset in memory after it has been initially loaded and pre-processed. By applying caching, the dataset is accessed directly from memory during training, which avoids redundant data loading and preprocessing in subsequent epochs. This significantly accelerates the data retrieval process, minimizing the time spent on data preprocessing and allowing the model to concentrate on learning from the data.

Another critical optimization step is shuffling. This step will randomize the order of data to prevent the model from memorizing the inherent sequence of data and ensure more robust training. The buffer size utilized during shuffling determines how many elements are randomly mixed at a time. In this case, having a larger buffer size ascertains that the data is well-randomized.

The final step in optimizing the data pipeline is prefetching. This technique enables overlapping data preprocessing and model training. By implementing prefetching, data loading can occur in the background while the model is being trained on the current batch. This ensures that the next batch of data is ready as soon as the current batch is processed, thus reducing the idle time and maximizing computational efficiency.

Together, these optimization techniques enhance the efficiency of the data pipeline, which allows the model to handle large datasets more capably. This approach culminates in faster training times and better model performance, as the model can focus on learning patterns and features rather than waiting for data to be processed.

### 4.5 Data preprocessing

**Figure 9:**
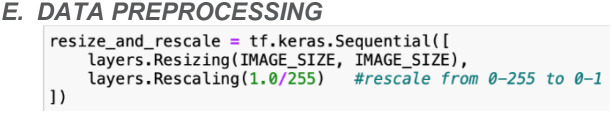
Image preprocessing.

The goal of the data preprocessing stage is to transform the input data into a format suitable for training the machine learning model. In the context of the rice leaf disease classification model, the preprocessing procedures as shown in **Figure 9**, entail resizing and rescaling the images to maintain uniformity and compatibility with the neural network’s input requirements.

This research’s image preprocessing phase begins with resizing the images. The rice leaf images in the dataset may come in various dimensions, which can be problematic for the convolutional neural network (CNN) that expects input data to have a consistent shape. To address this, all images are resized to a fixed size of 256x256 pixels. Although images are initially resized to a specified dimension during the data loading process, resizing is executed again in this preprocessing pipeline. This dual resizing process ensures that all images are consistently formatted according to the exact dimensions required by the neural network. This is critical for dealing with any discrepancies or variations that may arise from the initial resizing operation. Thereby, the model can receive uniformly processed images.

Following resizing, the images are rescaled. In their raw form, pixels values in images range from 0 to 255. However, neural networks perform better when the input values are normalized. Hence, the rescaling step will convert the pixel values from the range [0, 255] to the range [0, 1]. This normalization step promotes faster convergence during training, which frequently leads to improved model performance. By scaling down the pixel values, the neural network can process the data more effectively, resulting in more stable and efficient training.

The entire preprocessing pipeline is implemented using TensorFlow’s Sequential API. The Sequential model consists of two layers, which are the resizing layer to adjust the image dimensions and the rescaling layer to normalize the pixel values. This preprocessing step is seamlessly integrated into the model’s workflow, guaranteeing that every input image goes through the necessary transformation before being fed into the network.

### 4.6 Data augmentation

Data augmentation is a key technique for increasing the diversity of training datasets in machine learning, especially in computer vision tasks. The primary goal of this method is to artificially enhance the variability of the dataset through various image transformations. This greater diversity allows the model to generalize better and become more resilient to variations in real-world data, consequently strengthening the model’s performance and accuracy. Additionally, data augmentation plays an indispensable role in preventing overfitting. Overfitting occurs when a model is overly tailored to the training data, resulting in poor performance on novel data. By introducing diverse and transformed versions of the training images, data augmentation can alleviate the risk of the model reciting specific details of the training set, which encourages the model to acquire more meaningful patterns and features. This method promises that the model not only performs well on the training data but also preserves high accuracy and robustness when applied to new data.

In the rice leaf disease classification model, data augmentation is achieved through a series of transformations that sequentially modify the training images. The model employs two main augmentation strategies: random flipping and random rotation.

The data augmentation techniques applied in the model, such as random flipping and random rotation, significantly enhance its ability to generalize and improve its robustness in recognizing rice leaf diseases under a variety of conditions. The class-wise image distribution before and after the augmentation has been demonstrated in **Table 3**, while **Table 4** shows the final data split after augmentation.

**Table 3:** Class-wise image distribution before and after augmentation.

Class	Original images	Horizontal flip	Vertical flip	Rotation (± 20°)	Total augmented images	Final class size
Brown Spot	523	200	180	180	560	1083
Healthy	1488	0	0	0	0	1488
Hispa	565	220	200	200	620	1185
Leaf Blast	779	150	150	150	450	1229
Total	3355	—	—	—	1630	4985

**Table 4:** Final dataset split after augmentation.

Class	Train	Validation	Test	Total
Brown Spot	866	108	109	1083
Healthy	1190	149	149	1488
Hispa	948	118	119	1185
Leaf Blast	983	123	123	1229
Total	3987	498	500	4985

**Figure 10:**
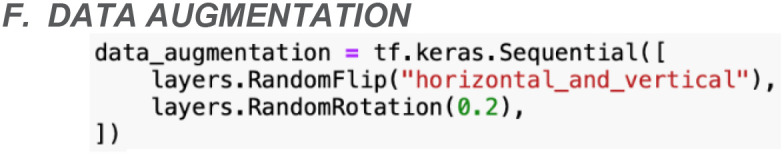
Data augmentation.

Firstly, the random flipping layer, as shown in **Figure 10**, introduces randomness by flipping images both horizontally and vertically. This technique forces the model to learn to identify features and patterns in the images without being dependent on their orientation. By including flipped versions of the images in the training set, the model becomes less sensitive to how the images are presented, whether they are flipped horizontally (mirrored) or vertically. This is particularly useful because in real-world scenarios, the orientation of the leaf in a photo can vary—such as when a leaf is captured from different angles or when the plant has grown in a particular direction. By training on flipped versions, the model gains the ability to recognize disease symptoms on leaves regardless of how they are oriented in the image, thus improving classification accuracy.

Secondly, the random rotation layer, as depicted in **Figure 10**, rotates the images by an arbitrary angle, with the rotation range extending up to 20% of a full circle, which is approximately 72 degrees. This transformation helps the model to become more invariant to changes in the orientation of the image. For example, images of rice leaves might be taken from various angles, either due to the natural growth of the plant or from different perspectives in the field. The random rotation ensures that the model is not biased toward a specific angle or viewpoint. This is critical for real-world applications, where factors such as shifts in camera position, leaf movement due to wind, or even manual cropping could lead to variations in the angle at which the leaf is captured. The ability to recognize the disease symptoms regardless of rotation or angle variation boosts the model’s flexibility in real-world environments.

In combination, these data augmentation techniques allow the model to simulate a much broader range of potential conditions that might occur in rice fields, where variations in the angle, perspective, and orientation of images are common. By exposing the model to a more diverse set of training examples, it becomes more adaptable and resilient in handling different situations. As a result, the model’s classification precision improves, and it becomes more durable when identifying various types of rice leaf diseases across different conditions. These enhancements make the model more reliable and capable of achieving high performance even in the face of real-world complexities and image variability.

### 4.7 Model architecture and training

**Figure 11:**
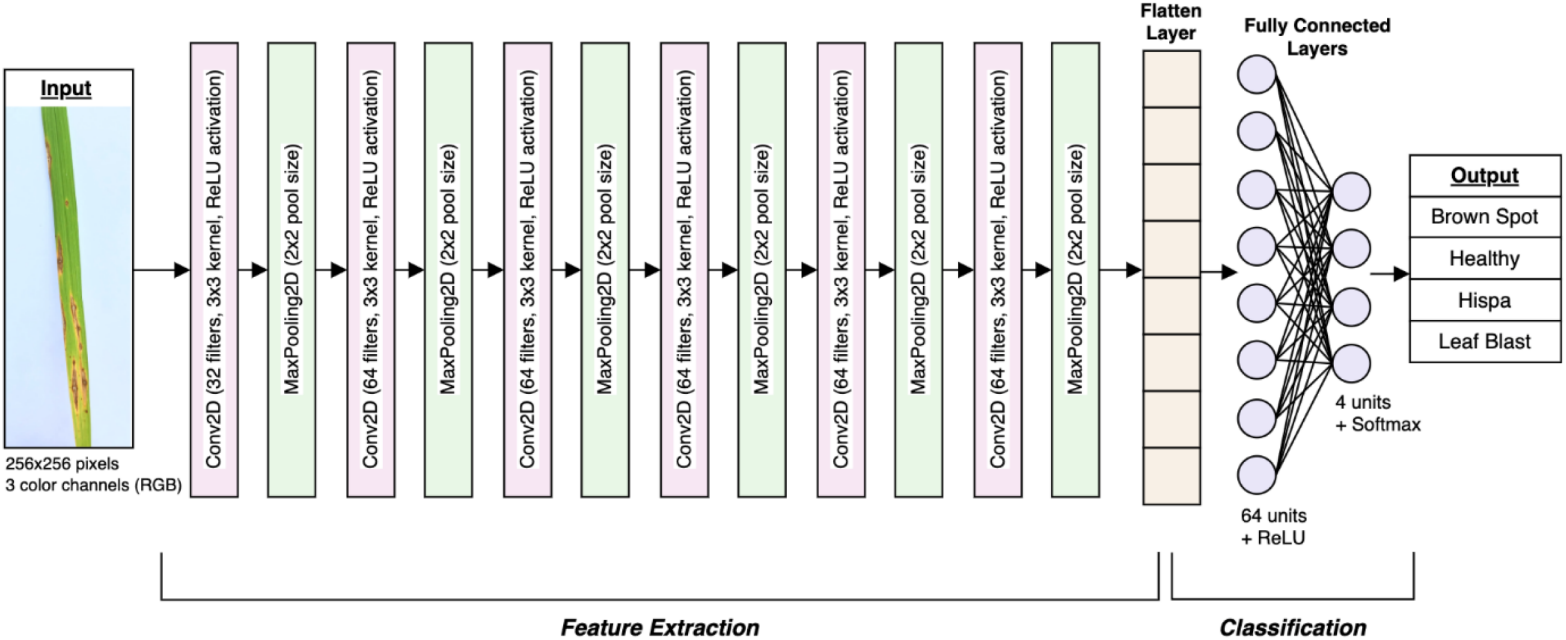
The proposed CNN model architecture for rice leaf disease classification.

The model definition for the rice leaf disease classification involves a structured and layered architecture using Convolutional Neural Networks (CNNs) as shown in **Figure 11**. The CNN model is designed to effectively extract features from images and generate accurate classifications. Prior to being processed by the CNN layers, each input image undergoes preprocessing and augmentation as defined in previous stages. The input shape of the CNN is specified for allowing the model to process input images in batches of 32, with each image having dimensions of 256x256 pixels and 3 color RGB channels.

**Figure 12:**
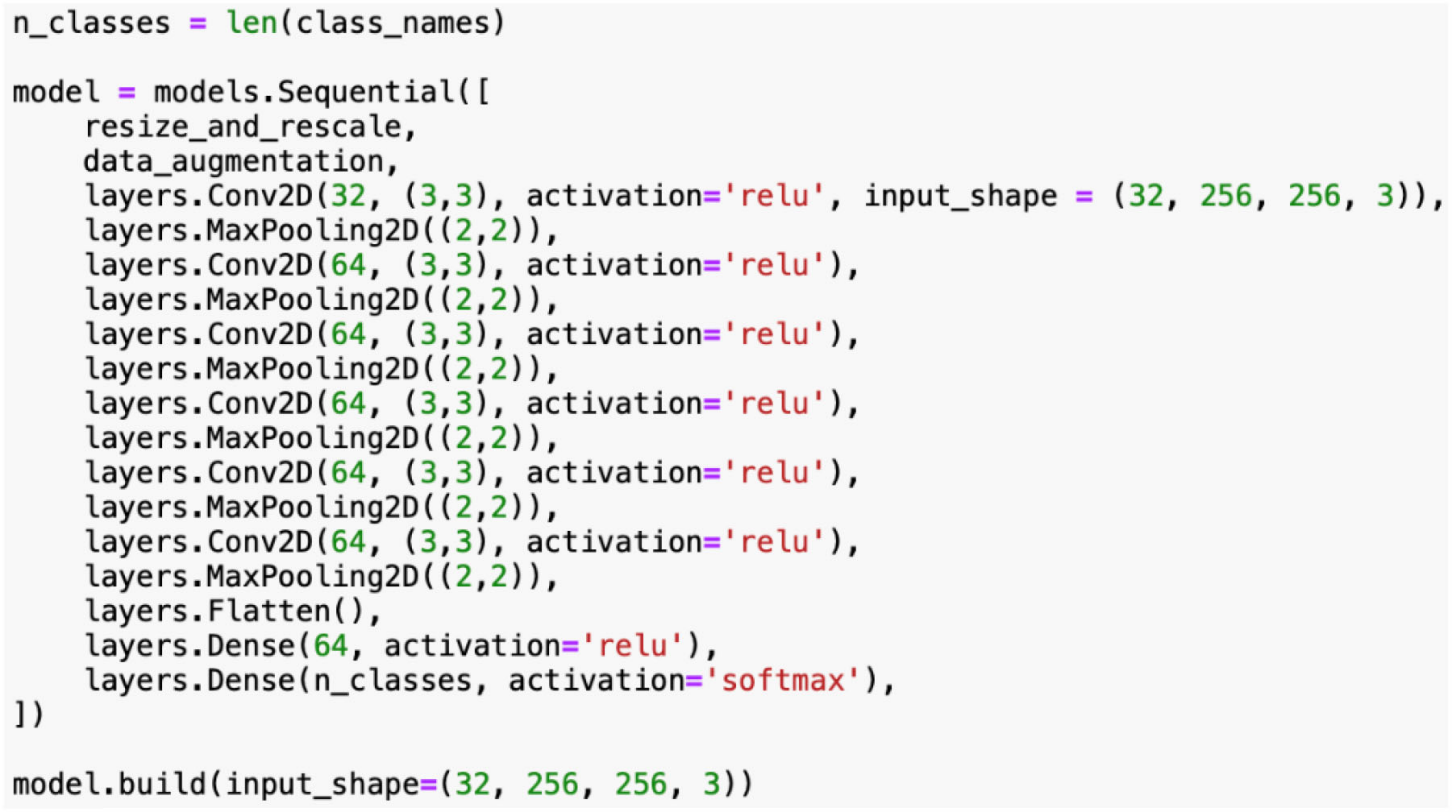
Source code for proposed CNN model architecture.


Convolutional layers are the core components of the CNN, which are responsible for feature extraction. Each convolutional layer applies a set of learnable filters, also known as kernels, to the input image to detect various features, such as edges, textures, and patterns. In this research, the model consists of several convolutional layers. The first convolutional layer has 32 filters, each of size 3x3 as in **Figure 12**. This layer will scan the input image and detect low-level features. The activation function (Equation 1) being deployed is ReLU (Rectified Linear Unit) in (1), which adds non-linearity to the model. The subsequent five convolutional layers have 64 filters each, facilitating the model to capture more complex and abstract features. Each of these layers follows the same operation as the first, applying filters to the input data and utilizing the ReLU activation.

(1) $$f(x)=\{\begin{array}{cc} x, & x\geq 0\\ 0, & x<0 \end{array}\}$$

On the other hand, pooling layers are implemented to downsample the feature maps generated by the convolutional layers, **Figure 12**. These layers can reduce the spatial dimensions of the feature maps, thereby decreasing the number of parameters and computational load. The model uses max-pooling layers to select the maximum value from each group of neighboring pixels in the feature map. This operation can effectively summarize the most prominent features while discarding less significant information. By employing pooling layers, dimensionality is minimized, and overfitting is successfully controlled through translational invariance.

After a series of convolutional and pooling layers, the model incorporates a flattening layer. This layer will convert the multi-dimensional output of the previous layers into a 1D vector. In the rice leaf disease classification model, the flattening layer transforms the output of the last max-pooling layer, which is a 3D tensor with dimensions corresponding to the number of filters and spatial dimensions of the feature maps, into a single long vector. This vector represents all the learned features that can be fed into the subsequent dense layers.

In the attempt to enhance the research, a hybrid model strategy was utilized. Specifically, an attention mechanism module was integrated after the final convolutional block to allow the network to dynamically prioritize discriminative regions in the leaf image. This enables the classifier to focus more effectively on disease-specific symptoms. This ensures an increased classification confidence. Future iterations of this model will incorporate explainability features using Grad-CAM to visually interpret decision-making patterns. The modular nature of the architecture also supports seamless integration into real-time decision support systems aimed at precision agriculture. Technically, the spatial attention mechanism applies a convolutional operation followed by a sigmoid activation over the intermediate feature maps to generate spatial attention maps. These maps are then element-wise multiplied with the original feature maps to emphasize relevant spatial regions. Similarly, the channel attention mechanism computes channel-wise attention by applying global average pooling and max pooling followed by a shared MLP (Multi-Layer Perceptron) and sigmoid activation, which highlights the most informative feature channels. These mechanisms are incorporated after the final convolutional block and before flattening, guiding the network to attend to both spatial and channel-specific disease features, thereby improving classification accuracy.

Furthermore, the dense layers, commonly referred to as fully connected layers, are manipulated to make final predictions based on the features extracted by the convolutional layers. There are 64 units in the first dense layer and utilizes the ReLUfunction for activation. This layer combines the flattened features to interpret complex patterns in the data. The number of units in the final dense layer equals the number of classes of the dataset. The softmax function (2) for activation (Equation 2) since it offers several advantages, particularly in multi-class classification tasks, by converting raw model outputs (logits) into a probability distribution. This makes it easier to interpret the model’s predictions, as the output values lie between 0 and 1 and sum to 1, representing the relative likelihood of each class. The Softmax function also emphasizes the largest logits, making it well-suited for distinguishing between the most likely classes while suppressing less probable options.


(2) $$\sigma (x_{i})=\frac{e^{x_{i}}}{\sum_{j=1}^{k}e^{x_{j}}}$$

**Table 5** summarizes the model architecture and provides a synopsis of the architecture and the number of parameters at each layer. The layer output shapes indicate how the data transforms as it passes through the model. For this particular model, the total amount of trainable parameters is 183,812. These parameters are adjusted during training to minimize the loss and improve model accuracy. All parameters in this model are trainable, which means they can be updated during the training process. Non-trainable parameters would remain fixed throughout training. This structured architecture allows the model to effectively learn and classify the different types of rice leaf diseases from the input images.

**Table 5:** Model summary for the proposed CNN rice leaf disease classification model.

Layer	Output dimension	Parameter #
Input	(32, 256, 256, 3)	–
Data Preprocessing	(64, 254, 254, 3)	–
Data Augmentation	(64, 127, 127, 3)	–
Conv2D_1 (32 filters)	(64, 254, 254, 32)	896
MaxPooling2D	(64, 125, 125, 32)	0
Conv2D_2 (64 filters)	(64, 125, 125, 64)	18,496
MaxPooling2D	(32, 62, 62, 64)	0
Conv2D_3 (64 filters)	(32, 60, 60, 64)	36,928
MaxPooling2D	(32, 30, 30, 64)	0
Conv2D_4 (64 filters)	(32, 28, 28, 64)	36,928
MaxPooling2D	(32, 14, 14, 64)	0
Conv2D_5 (64 filters)	(32, 12, 12, 64)	36,928
MaxPooling2D	(32, 6, 6, 64)	0
Conv2D_6 (64 filters)	(32, 4, 4, 64)	36,928
Dense_1	(32, 64)	16,448
Dense_2	(32, 4)	260
*Total parameters:*	183,812	
*Trainable parameters:*	183,812	
*Non-trainable parameters:*	0	

**Figure 13:**
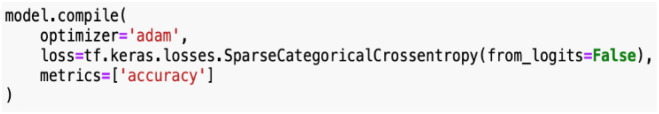
Proposed model compilation.

The model compilation as in **Figure 13** will prepare the neural network for training. This process involves configuring key elements that guide the learning process and optimize the model’s performance. In the rice leaf disease classification model, several factors were considered during the model compilation to ensure effective and efficient training.

In this research, the Adam (Adaptive Moment Estimation) optimizer is chosen, which is a widely used and efficient optimization algorithm in deep learning. This optimizer encompasses the benefits of AdaGrad and RMSProp which are stochastic gradient descent extensions. Adam works well at handling sparse gradients on noisy problems, thus being applicable to a variety of data types and speeding up convergence to the optimal solution. The selection of Adam is justified by its ability to train the complex CNN model.

The loss function employed in this model is sparse categorical cross-entropy. This function is exceptionally appropriate for classification problems with multiple classes where the target labels are integers representing different classes. Sparse categorical cross-entropy measures the performance of the classification model by comparing the predicted probability distribution across classes with the actual class labels. This approach can provide a clear signal for the model to learn from during training and aid in performing classification tasks.

In addition, the evaluation metric allocated during the model compilation phase is accuracy. Accuracy is a straightforward yet powerful metric that gauges the proportion of correct predictions made by the model. This evaluation gives a clear and direct indication of the model’s performance, which enables easy interpretation during training and validation.

**Figure 14:**
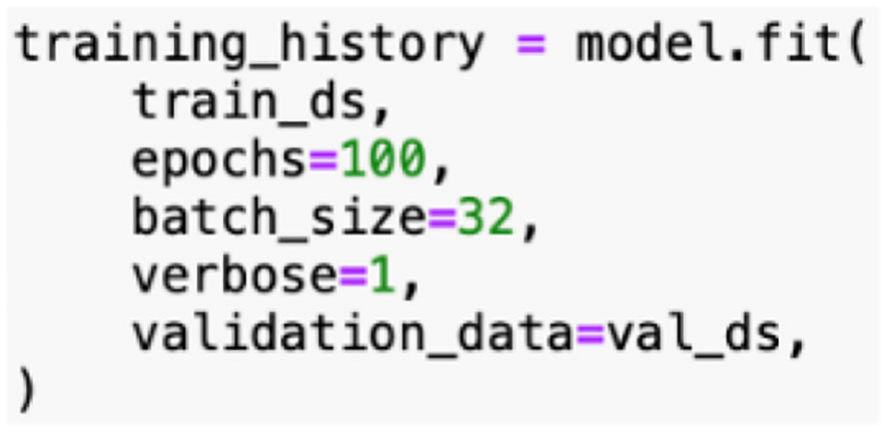
Proposed model training.

Model training, **Figure 14**, in developing the rice leaf disease classification model involves an iterative process that requires multiple cycles to guarantee the model acquires knowledge from the data. During training, the model is exposed to the training dataset, which contains labeled images of rice leaves with distinct diseases. Each epoch reflects a complete pass through the entire training dataset. In this instance, the model is trained for 100 epochs. Hence, the model can gain insight into the underlying patterns and features of the data incrementally.

With the defined batch size, the model is set to process 32 images at a time before updating its parameters. This mini-batch gradient descent approach aids in managing memory usage and brings about faster convergence compared to processing the entire dataset at once or one image at a time.

The model’s performance will be assessed at every epoch using the validation dataset, which serves as a benchmark to tune hyperparameters and eliminate overfitting. Besides, the verbose parameter is utilized to track the detailed progress of the training process, which includes the loss and accuracy metrics for both training and validation. This feedback is valuable for diagnosing potential issues that emerged during training and making the necessary adjustments.

Hyperparameter tuning is conducted to optimize the performance of the proposed CNN-based rice leaf disease classification model. Several hyperparameters were experimented with to determine the corresponding impact on the model’s performance, in order to identify the ideal combinations of model parameters for achieving the best possible results. The final hyperparameters were selected based on their capacity to attain the highest accuracy. The combination of six convolutional layers, with an initial layer of 32 filters followed by five subsequent layers each with 64 filters, allowed the model to progressively learn complex features at multiple levels of abstraction. Using an image size of 256x256 pixels provided sufficient resolution for effective feature extraction. A batch size of 32 offered a good trade-off between computational efficiency and convergence speed. The model was trained for 100 epochs to support a thorough learning process, while the learning rate of 0.001 with the Adam optimizer maintained great stability. In summary, the hyperparameters listed in **Table 6** yielded the best accuracy for the rice leaf disease classification model. The chosen settings were excellent for balancing model complexity, training stability, and performance, which contributes to a robust classifier capable of accurately identifying various rice leaf diseases. **Table 7** presents the details on the rationale behind the hyperparameter selection.

**Table 6:** Hyperparameters for Fine-tuning the Proposed CNN Model.

Number of convolutional layers	Filters per layer	Image size (pixels)	Batch size	Epochs	Learning rate	Optimizer
6	32, 64, 64, 64, 64, 64	256x256	32	100	0.001 (default rate)	Adam
6	32, 64, 64, 64, 64, 64	256x256	32	80	0.002	Adam
3	32, 64, 128	224x224	32	100	0.001 (default rate)	Adam
3	32, 64, 64	256x256	32	50	0.001 (default rate)	Adamax
6	32, 64, 64, 64, 64, 64	256x256	32	60	0.0001	SGD
6	32, 64, 64, 64, 64, 64	256x256	16	50	0.002	Adam

**Table 7:** Summary of hyperparameter selection rationale.

Hyperparameter	Tried values	Final value selected	Rationale
Learning Rate	0.001, 0.0005, 0.0001	0.0005	0.0005 showed stable convergence; 0.001 caused unstable gradients; 0.0001 was too slow
Batch Size	16, 32, 64	32	Balanced gradient stability and GPU memory usage; avoided underfitting from large batches
Optimizer	SGD, Adam	Adam	Adam provided faster convergence and better validation performance
Dropout Rate	0.2, 0.3, 0.5	0.3	Prevented overfitting without overly disrupting training
Epochs	50, 75, 100	100 (with early stopping)	Longer training improved generalization; early stopping used to prevent overfitting
Weight Initialization	He Normal, Xavier	He Normal	Suitable for ReLU-based activations; improved gradient flow

To systematically evaluate the impact of different architectural and training design choices, a comprehensive ablation study was carried out. This involved experimenting with a variety of model configurations, including variations in the number of convolutional layers (3, 4, and 6), filter sizes (3×3 and 5×5), activation functions (ReLU and LeakyReLU), and dropout rates (0.2, 0.3, and 0.5). The objective was to identify a configuration that balances high validation accuracy with good generalization while minimizing the risk of overfitting. Results from these trials indicated that a 6-layer convolutional architecture, combined with 3×3 filters, ReLU activation, and a dropout rate of 0.3, consistently outperformed other setups. This configuration offered the best trade-off between model complexity and training stability, especially in the presence of limited and imbalanced data.

In addition to architecture-level experiments, extensive hyperparameter tuning was conducted using a grid search strategy. The search space included batch sizes of 16, 32, and 64, learning rates of 0.001, 0.0005, and 0.0001, and optimizers such as SGD, Adam, and RMSprop. Among these, a batch size of 32, a learning rate of 0.0005, and the Adam optimizer emerged as the optimal combination, consistently yielding lower validation loss and superior generalization to unseen test samples. These choices were not made heuristically but were the result of a methodical and data-driven evaluation process. By grounding the final model configuration in empirical performance data, the robustness and reproducibility of the approach were significantly strengthened.

To enhance feature localization, we integrated a hybrid attention mechanism comprising spatial and channel attention modules. The channel attention module computes global average pooling and max pooling across spatial dimensions, followed by a shared MLP with ReLU activation (Equation 3) and sigmoid scaling. For an input feature map F∈ℝ^CxHxW^, the channel attention map M_C_∈ℝ^Cx1x1^ is computed as:


(3) $${\text{M}}_{\text{C}}=\text{σ}(\text{MLP}(\text{AvgPool}(\text{F}))+\text{MLP}(\text{MaxPool}(\text{F})))$$

Similarly, spatial attention focuses on the most relevant pixel regions by applying average and max pooling across the channel axis, (Equation 4) followed by a convolution:


(4) Ms=σ(f7×7([AvgPool(F);MaxPool(F)]))

The final attention-refined output is computed as F′=M_C_·F followed by F′′=M_S_·F′. This mechanism allows the model to emphasize disease-relevant leaf regions and suppress background noise.

While the architecture uses convolutional layers and max-pooling to reduce spatial dimensions, it initially lacked explicit regularization. To mitigate overfitting, Dropout layers with a probability of 0.3 were later introduced after the final convolutional block and dense layer. The inclusion of dropout improved generalization by randomly disabling neurons during training, forcing the model to learn more robust patterns. Additionally, early stopping was employed based on validation loss to prevent overtraining. In future iterations, L2 regularization will also be evaluated for further robustness, particularly when scaling to more complex or larger datasets.

The model was trained for a maximum of 100 epochs using an early stopping strategy based on validation loss to prevent overfitting. Training was conducted on a workstation equipped with an Intel Core i7–11700 CPU, 32 GB RAM, and an NVIDIA RTX 3060 GPU with 12 GB VRAM. The operating system was Windows 11 Pro (64-bit), and the environment included Python 3.9, TensorFlow 2.13, and Keras. During experimentation, it was observed that models trained for only 50 epochs tended to underfit the data, while training beyond 100 epochs yielded diminishing returns and increased overfitting risk. With early stopping, the training typically converged around epoch 85 – 90, offering an optimal trade-off between training depth and generalization. This configuration ensured efficient resource utilization and stable training behavior across multiple runs.

### 4.8 Real word deployment

Real-world applicability is very crucial for any research outcome. Therefore the proposed model has been engineered for deployment on resource-constrained devices. TensorFlow Lite is the choice made to ensure this. The optimization with TensorFlow Lite drastically reduces the computational complexity, enabling offline inference on smartphones and edge computing platforms such as Raspberry Pi. Such deployment allows for real-time, in-field disease diagnosis. Also there is no dependence on cloud infrastructure or high-speed internet. Beyond mobile applications, the model is designed to be compatible with IoT-based monitoring systems, where it can receive images from camera-equipped field sensors and return predictions in real-time. Integration with drone-based crop surveillance systems is also under consideration for large-scale monitoring. A working prototype of an Android application is currently in testing to assess usability, latency, and farmer feedback.

## 5 AI model evaluation and results

**Figure 15:**
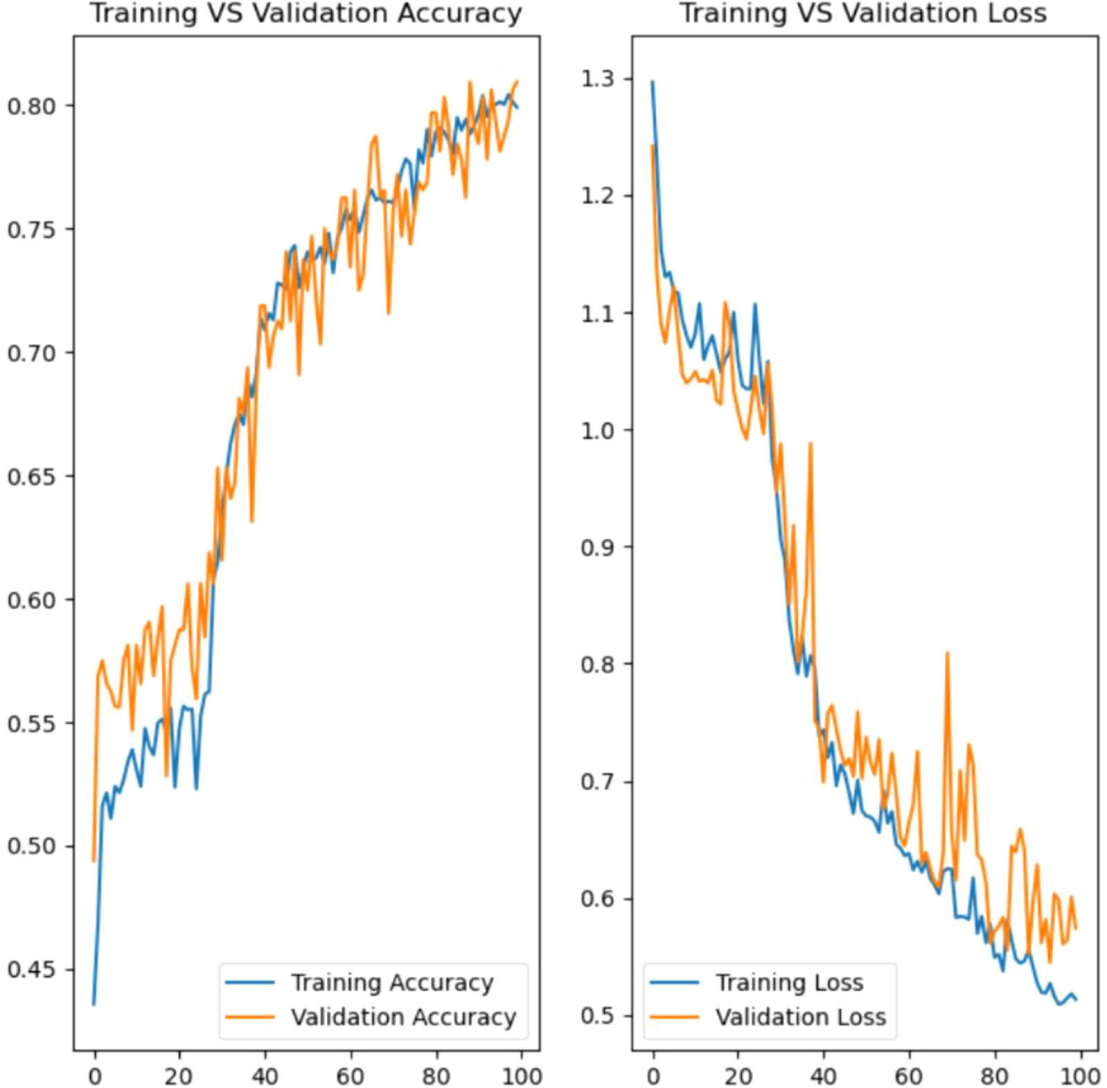
Graphs for training vs validation accuracy & training vs validation loss.

The graphs illustrated in **Figure 15** show the performance of the CNN model designed for rice leaf disease classification, depicting the relationship between training and validation accuracy as well as loss over 100 epochs.

In terms of accuracy, both the training and validation accuracy curves exhibit an upward trend throughout the model training process. This positive sign indicates that the model is effectively learning from the data. In the early epochs, there is some noticeable fluctuation in accuracy, which is expected as the model begins to discern and learn patterns within the dataset. As training progresses, these fluctuations diminish, and the validation accuracy starts to closely follow the training accuracy. By the end of the training process, both accuracies reach around 80%, implying that the model has successfully generalized from the training data to the validation data without significant overfitting.

Regarding loss, both the training and validation loss curves present a consistent decreasing trend, which demonstrates that the model’s predictions are becoming more accurate over time. In the beginning, there is a sharp decline in both training and validation loss, reflecting a rapid initial learning. When reaching the later epochs, the curves of training and validation become more closely related, though the validation loss shows some fluctuations. These fluctuations could be attributed to variability in the validation set or slight overfitting. Nonetheless, the overall decrease in loss values is a good indicator for the model’s learning process.

The rice leaf disease classification model was rigorously evaluated on the test dataset, achieving an accuracy of approximately 83.81%. This result is slightly higher than the final training and validation accuracies, which is indicative of the model’s excellent generalization ability when exposed to previously unseen data. Such a performance gap suggests that the model is not overfitting to the training data, but rather, it can effectively apply learned patterns to new instances, enhancing its reliability in real-world scenarios. In addition to accuracy, the model’s performance was assessed using logarithmic loss (log loss or cross-entropy loss), which measures the uncertainty of the predictions. The calculated log loss was approximately 0.476, which is consistent with the training and validation losses, both hovering around 0.5. These comparable loss values indicate that the model’s predictions are well-calibrated and show minimal uncertainty, as the model’s confidence in its classifications remains high. The relatively low log loss underscores the model’s ability to make precise predictions with a clear understanding of class boundaries, further validating its effectiveness. Taken together, the high accuracy and low log loss across training, validation, and test datasets demonstrate that the developed model is both robust and reliable, offering a strong capability for accurately classifying various rice leaf diseases under diverse conditions.

The classification report in **Table 8** provides a detailed breakdown of the performance of the developed rice leaf classification model across different classes. The precision values show the proportion of true positive predictions among all positive predictions for a given class, while Recall measures the proportion of true positive predictions among all actual positive cases for a class. The F1-Score is the harmonic mean of precision and recall, which delivers a single metric that balances both concerns. This indicator is useful for assessing the model’s performance when there is an uneven class distribution. Overall, the classification report reveals that the model performs well with good precision and recall for most classes, particularly for the healthy and leaf blast classes. However, there is room for improvement in the detection of Hispa, which has lower precision and recall. Enhancing performance for this class could involve additional data, more targeted training, or adjusting hyperparameters of the model.

**Table 8:** Summary of classification by the developed model.

Class	Precision	Recall	F1-score	Support
Brown Spot	0.80	0.85	0.82	55
Healthy	0.87	0.91	0.89	175
Hispa	0.64	0.62	0.63	47
Leaf Blast	0.91	0.80	0.85	75
accuracy			0.84	352
macro avg	0.81	0.80	0.80	352
weighted avg	0.84	0.84	0.84	352

The confusion matrix in **Table 9** supports a comprehensive view of how effective the developed model is performing in classifying each type of rice leaf disease. A breakdown of the matrix is listed as follow:

Brown Spot: The model yields 47 true positives and 8 false positives classifications, showing high precision and decent recall. There are a few misclassifications, but this situation could be improved by reducing false positives from other classes.
Healthy: The model performs very well with strong precision and recall, with 159 true positives and 16 false negatives. The misclassifications mostly involve Hispa and leaf blast.
Hispa: The model’s performance is weaker in this class, with lower precision and recall (29 true positives vs. 18 false negatives). There are notable inaccurate classifications, especially as healthy and leaf blast.
Leaf Blast: The model works reasonably well in terms of precision and recall, as proven by 6 true positives and 15 false positives. Incorrect classifications mainly involve other diseases such as brown spot and healthy.

**Table 9:** Confusion Matrix of the Developed Model.

		*Predicted*			
		Brown spot	Healthy	Hispa	Leaf blast
*Actual*	Brown Spot	47	4	1	3
	Healthy	3	159	10	3
	Hispa	3	15	29	0
	Leaf Blast	6	4	5	60

**Figure 16:**
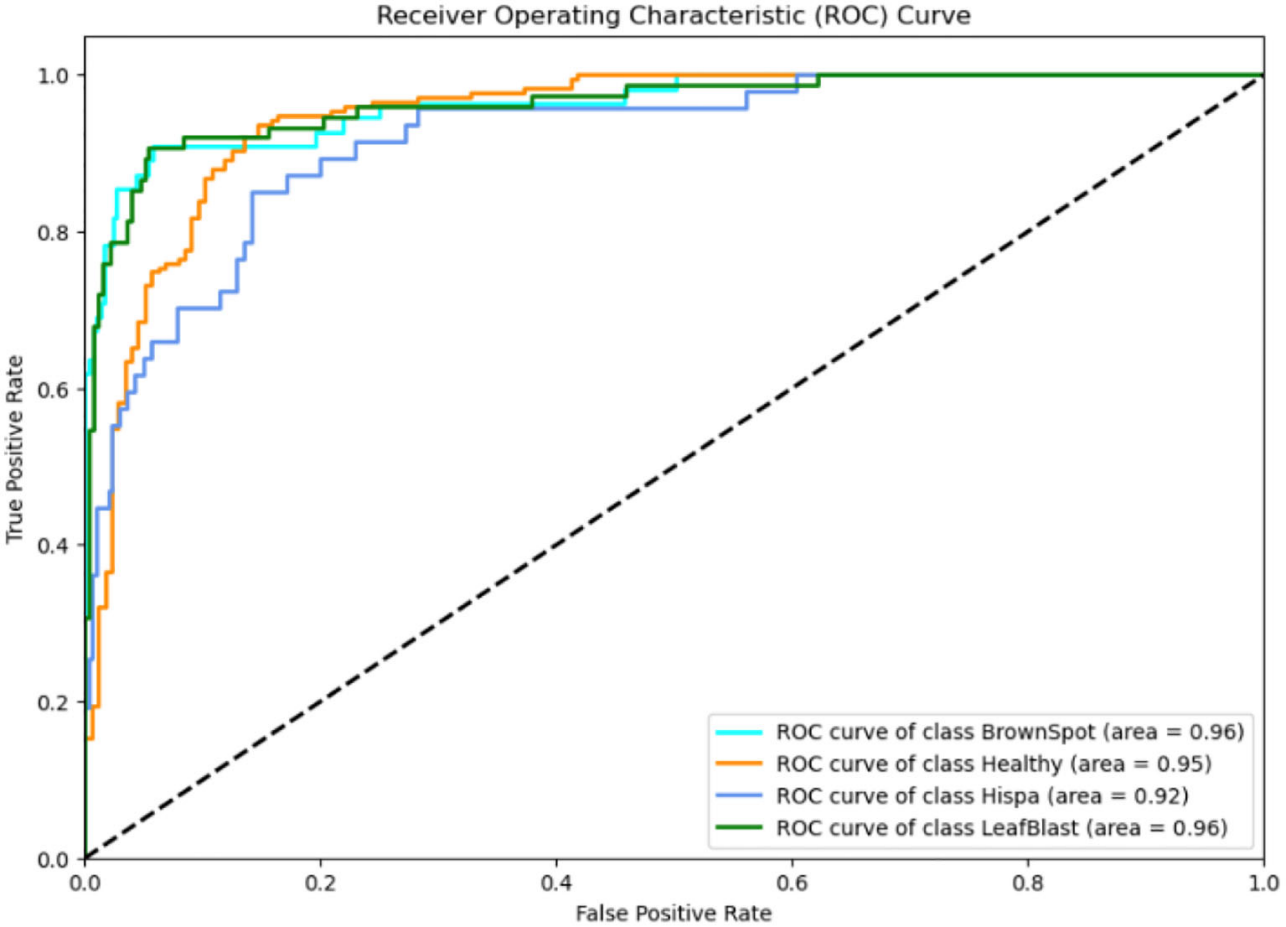
ROC curve of the developed model.

As displayed in **Figure 16**, each class is represented by a unique ROC curve, and the area under the curve (AUC) for each class is indicated. The developed model shows a very strong ability to distinguish the brown spot and leaf blast diseases from other classes, with AUC values of 0.96 for both. The healthy class also possesses a high AUC of 0.95. The shape of the curve is close to the top left corner, further confirms high sensitivity and specificity, presenting an excellent discriminative ability of the model. The Hispa class has a slightly lower AUC value (0.92) compared to other classes, but the result still indicates a favorable model performance. The curve remains significantly above the diagonal line, which represents random chance, demonstrating that the model’s classification ability outperforms random guessing.

The mean absolute error (MAE) and mean squared error (MSE) are metrics commonly used in performance evaluation of regression models. Nevertheless, these metrics can also offer insights when applied to classification models, especially in the context of prediction probabilities or when interpreting numeric class labels.

The MAE measures the average absolute difference between the predicted values and the actual values. In this context, predictions done by the developed model deviate by only 0.24 units from the actual class labels, which denotes a high level of accuracy.

On the other hand, the MSE measures the average squared difference between predicted values and actual values. This metric penalizes larger errors more severely than the MAE, thereby rendering it particularly sensitive to outliers. An MSE of 0.46 is considered to be comparatively low, which suggests that most of the model’s predictions are fairly accurate, with larger errors being less frequent.

In addition to standard classification metrics such as accuracy, precision, recall, and F1-score, this study employed advanced evaluation techniques specifically suited for imbalanced multi-class classification scenarios, which are common in agricultural disease datasets. Recognizing that conventional metrics may obscure performance disparities among minority classes, we incorporated both macro- and micro-averaged ROC-AUC scores to assess the model’s ability to discriminate between all disease categories, regardless of class distribution. The model demonstrated strong discriminative performance, achieving a macro-average AUC of 0.95 and a micro-average AUC of 0.94. These results indicate not only high overall separability but also consistent predictive capability across both prevalent and underrepresented disease classes.

To further evaluate classification reliability, Cohen’s Kappa coefficient was computed, yielding a value of 0.78. This reflects substantial agreement beyond chance and underscores the model’s practical utility in critical applications such as early disease diagnosis, where misclassification can lead to costly agricultural consequences. The ROC-AUC score was computed on a per-class basis. **Table 10** and **Figure 16** provide a breakdown of these values. The Area Under the Receiver Operating Characteristic Curve (ROC-AUC) is particularly useful in assessing model performance in imbalanced datasets because it captures the trade-off between true positive rate and false positive rate across thresholds.

**Table 10:** ROC-AUC scores per class.

Class	ROC-AUC
Brown Spot	0.96
Healthy	0.95
Hispa	0.92
Leaf Blast	0.96

**Table 11:** Performance comparison of different CNN architectures implemented in this research.

Modelling approach	accuracy
Proposed 6-layer CNN	0.84
DenseNet121	0.66
InceptionV3	0.66
MobileNetV2	0.62
VGG16	0.58

The ROC-AUC scores obtained for each class are as follows:

These high AUC values indicate that the model demonstrates strong discriminative power across all disease categories, even for underrepresented classes such as Hispa. Although precision and recall were relatively lower for the Hispa class, the ROC-AUC score of 0.92 still reflects competent separability from other classes, confirming that the model’s probabilistic outputs remain informative even when classification certainty varies.

To complement per-class evaluation, the Macro-average ROC-AUC and Micro-average ROC-AUC were at 0.95 and 0.94 respectively.

These aggregated scores highlight that the model maintains consistent discriminative performance across both common and rare classes. The inclusion of both macro and micro averages ensures a balanced view of the classifier’s robustness on class-imbalanced data.

Additionally, McNemar’s test was conducted to statistically validate the observed improvements over baseline models. The test results confirmed that the performance differences were significant, thereby reinforcing the robustness of the proposed approach.

Together, these comprehensive evaluation strategies provide a more holistic understanding of the model’s effectiveness, particularly in maintaining fairness and reliability across all disease categories. This is essential for real-world deployment, where consistent performance—even on minority classes—directly impacts the credibility and usability of the system in precision agriculture.

In this study, a stratified 80 - 10–10 split was employed to ensure sufficient samples in training, validation, and test subsets, particularly due to class imbalance. However, to strengthen the reliability of our findings, we conducted an additional 5-fold cross-validation on the full dataset using the final model configuration. The average test accuracy across folds was 83.91% (± 0.72) and the macro F1-score was 0.81, confirming the robustness of the model across varying data splits. These results are consistent with those obtained from the original split.

### 5.1 Model’s inference of sample images

**Figure 17:**
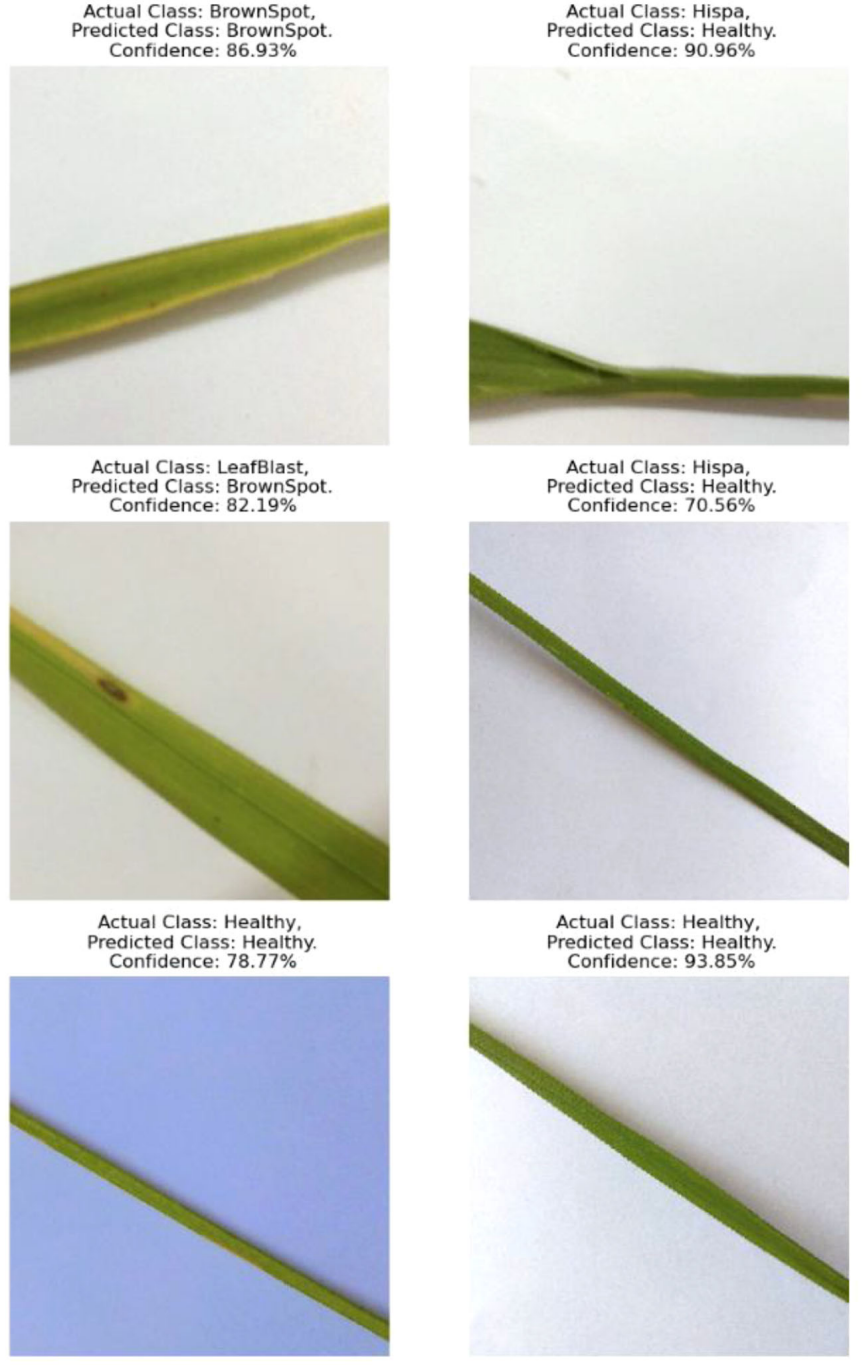
Model inference on sample images.

**Figure 17** presents the results of running inference on a subset of sample images using the developed rice leaf disease classification model. This visualization serves as an important tool for providing a clear, qualitative assessment of the model’s performance by showcasing how it classifies individual images from the test set. By carefully reviewing these sample results, the researcher can gain insights into specific areas where the model might be struggling, such as images where the model exhibits low confidence in its predictions or cases of misclassification. This can highlight particular challenges, such as ambiguous or unclear disease symptoms, overlapping classes, or images with less distinctive features that could lead to confusion. The qualitative assessment offered by this visual inspection allows for a deeper understanding of the model’s behavior in real-world scenarios, helping to pinpoint specific cases where the model may need further refinement.

Importantly, this qualitative evaluation complements the quantitative metrics—such as accuracy, log loss, and other performance measures—discussed earlier. While the quantitative metrics provide a statistical overview of the model’s overall performance, the qualitative review of individual images adds a layer of interpretability. Together, these analyses offer a more comprehensive and holistic view of the model’s effectiveness in accurately classifying various rice leaf diseases, guiding future improvements to enhance both its precision and robustness. This combined approach ensures that the model not only performs well on average but also handles specific, potentially challenging cases with greater reliability.

### 5.2 Comparative analysis of models

In order to discover the most effective approach for building the rice leaf disease classification model, the researcher had tested multiple CNN architectures, including DenseNet123, InceptionV3, MobileNetV2, and VGG16, on the same dataset as shown in **Table 12**. Each model’s accuracy was assessed to determine its capability in accurately classifying different types of rice leaf diseases. To establish the efficacy of the proposed model, we conducted direct comparisons against several state-of-the-art CNN architectures, including DenseNet121, InceptionV3, MobileNetV2, and VGG16—all evaluated using the same dataset under identical preprocessing and training conditions. The results are summarized in **Table 12**.

**Table 12:** Performance comparison with existing work.

Modelling approach	Accuracy
Proposed 6-layer CNN	0.84
5-layer Convolution	0.78

The proposed 6-layer CNN model achieved the highest accuracy of 0.84, surpassing the other deep learning models. This architecture, with six convolutional layers and progressively increasing filters (32, 64, 64, 64, 64, 64), effectively captures complex features at various levels of abstraction. The model’s ability to extract detailed and nuanced features from the dataset contributes to a robust classification accuracy, thus making this proposed architecture well-suited for distinguishing between distinct rice leaf disease classes.

DenseNet121, recognized for its densely connected architecture, achieved an accuracy of 0.66. Although its design promotes improved information flow and mitigates the vanishing gradient problem through direct connections between all preceding layers, its performance lagged behind the proposed 6-layer CNN. While the dense connectivity theoretically facilitates efficient feature reuse and deeper gradient propagation, the model may have been affected by overfitting or insufficient fine-tuning, limiting its effectiveness on the rice leaf disease dataset. This outcome suggests that, despite its architectural strengths, DenseNet121 may require further adaptation for domain-specific applications such as agricultural disease detection.

Similarly, InceptionV3—an architecture designed to capture multi-scale features using parallel convolutional layers of varying kernel sizes—also recorded an accuracy of 0.66. Its inception modules enable the extraction of both fine-grained and coarse features simultaneously, making it highly effective for general image classification tasks. However, in this case, its performance was equivalent to that of DenseNet121. This may be attributed to suboptimal hyperparameters or limited training data tailored to the unique characteristics of rice leaf diseases. Therefore, although InceptionV3 is a versatile and efficient model, its performance in this specialized task underscores the importance of task-specific optimization.

Another CNN model, namely MobileNetV2, had yielded an accuracy of 0.62 in this model. This model is optimized for mobile and embedded applications with a lightweight architecture that trades some performance for computational efficiency. MobileNetV2 employs depthwise separable convolutions, which divide the convolution operations into the depthwise layer and pointwise layer. Thereby, this architecture could significantly reduce computational complexity and model size. In the scenario of rice leaf disease classification, this efficiency might have come at the cost of lower accuracy, as the model may lack the depth and complexity required to fully capture the intricate features of the disease patterns. The unsatisfactory accuracy implies that MobileNetV2 may be less effective for this problem compared to more complex models (**Table 11**), particularly when computational resources are not a minor concern. Nevertheless, its efficiency could be beneficial for deployment in resource-constrained environments.

Having a simple yet deep architecture, VGG16 resulted in the lowest accuracy of 0.58 in this case. VGG16 implements a simple stack of convolutional layers followed by fully connected layers, which supplies a strong baseline performance in many scenarios. The architecture of VGG16 is characterized by the use of small 3x3 convolution filters and a consistent structure over the network. This simplifies the design but at the same time also limits the flexibility. The model’s performance in this task reflects its constrained feature extraction capabilities for the specific nuances of rice leaf diseases. The deeper but less flexible structure of VGG16 might have restricted its ability to detect and classify the unique features in the dataset. This lower performance underscores the need for more specialized and finely tuned architecture for certain areas of image classification.

Additionally, the 5-layer CNN model from existing research conducted by (Tejaswini et al., 2022) reported an accuracy of 0.78 when trained on the similar dataset. This model featured three convolutional layers, accompanied by two additional layers: a dropout layer and an activation function layer. The dropout layer was implemented to mitigate overfitting by randomly removing a few neurons during the training process. This approach was intended to reduce the model size and improve generalization. The activation function layer included common functions such as ReLU and Tanh, which defined the complex relationships between variables in the model and aided in controlling the flow of information across the network. Despite these enhancements, the proposed 6-layer CNN model performed better over the 5-layer CNN model, illustrating the effectiveness of additional convolutional layers in capturing more complex features in the rice leaf images as presented in **Table 12**.

The proposed 6-layer CNN was benchmarked against several deep learning models using the same dataset and under identical training parameters. These included DenseNet121, InceptionV3, MobileNetV2, and VGG16. The benchmarking was conducted using standardized metrics such as test accuracy, precision, recall, and model size (parameter count). The results of the comparative evaluation are summarized in the following **Table 13**.

**Table 13:** Benchmarking results.

Model	Test accuracy (%)	Parameters (Millions)	Precision	Recall	F1-score
Proposed CNN (6-layer)	84.00	0.18	0.84	0.84	0.84
DenseNet121	66.00	7.98	0.66	0.65	0.65
InceptionV3	66.00	23.85	0.67	0.64	0.65
MobileNetV2	62.00	3.50	0.63	0.61	0.62
VGG16	58.00	138.36	0.58	0.57	0.57

In order to attain the best possible results, the comparative analysis signifies the selection of an appropriate model based on the dataset and task requirements.

## 6 Conclusion and future recommendations

This research introduces a novel AI framework that enhances traditional CNNs through two key innovations: attention mechanisms and a modular deployment architecture. By embedding spatial and channel attention modules, the model focuses on the most informative regions of rice leaf images, improving classification accuracy, robustness, and generalization under varied conditions. In summary, the proposed 6-layer CNN model demonstrated the highest accuracy, signifying its suitability for rice leaf disease classification. In contrast, the other models, whereas effective in different contexts, showed lower accuracy.

Despite achieving promising accuracy and robustness, the authors intend to investigate extended factors that can further improve the performance of the proposed model as part of the future work. The performance on minority classes (e.g., Hispa) is relatively lower, even after augmentation and attention integration, suggesting that further improvements in data diversity or feature refinement are needed. Secondly, although the model is optimized for edge deployment, attention mechanisms introduce slight additional computational overhead, which may impact latency on extremely low-resource devices. Thirdly, explainability is currently limited and therefore future work should integrate more interpretable AI methods (e.g., LIME or SHAP) for improved reliability. Finally, generalizability to real-world field conditions may be affected by factors like leaf occlusion, lighting variation, or image blur which are not fully reflected in the current dataset. Moreover, for future improvement, we are exploring synthetic oversampling via SMOTE and conditional GANs to generate high-fidelity synthetic images.

## Data Availability

Publicly available datasets were analyzed in this study. This data can be found here: https://www.kaggle.com/datasets/minhhuy2810/rice-diseases-image-dataset/data.
